# Bacteria-based immunotherapy for cancer: a systematic review of preclinical studies

**DOI:** 10.3389/fimmu.2023.1140463

**Published:** 2023-08-03

**Authors:** Min Zhou, Yucheng Tang, Wenjie Xu, Xinyan Hao, Yongjiang Li, Si Huang, Daxiong Xiang, Junyong Wu

**Affiliations:** ^1^ Department of Pharmacy, The Second Xiangya Hospital, Central South University, Changsha, China; ^2^ Hunan Provincial Engineering Research Centre of Translational Medicine and Innovative Drug, Changsha, China; ^3^ Institute of Clinical Pharmacy, Central South University, Changsha, China; ^4^ Hunan Key Laboratory of Tumor Models and Individualized Medicine, The Second Xiangya Hospital, Changsha, China

**Keywords:** bacteria, immunotherapy, cancer, tumor model, vaccine, colonization

## Abstract

Immunotherapy has been emerging as a powerful strategy for cancer management. Recently, accumulating evidence has demonstrated that bacteria-based immunotherapy including naive bacteria, bacterial components, and bacterial derivatives, can modulate immune response *via* various cellular and molecular pathways. The key mechanisms of bacterial antitumor immunity include inducing immune cells to kill tumor cells directly or reverse the immunosuppressive microenvironment. Currently, bacterial antigens synthesized as vaccine candidates by bioengineering technology are novel antitumor immunotherapy. Especially the combination therapy of bacterial vaccine with conventional therapies may further achieve enhanced therapeutic benefits against cancers. However, the clinical translation of bacteria-based immunotherapy is limited for biosafety concerns and non-uniform production standards. In this review, we aim to summarize immunotherapy strategies based on advanced bacterial therapeutics and discuss their potential for cancer management, we will also propose approaches for optimizing bacteria-based immunotherapy for facilitating clinical translation.

## Introduction

Traditional cancer therapies focus on inhibiting tumor growth while largely ignoring the role of the immune system in killing cancer cells (1). In the last ten years, numerous ground-breaking immunotherapies have been authorized for clinical practice (2). Especially cancer vaccines and immune checkpoint inhibitors (ICIs) (3). However, there is still no effective strategy to implement a cure since the hypoxia and immunosuppressive tumor microenvironment significantly restrict antitumor immunity (3). Additional research demonstrated that bacteria activate immune cells even in the immunosuppressive microenvironment of tumors. The rich pathogen-associated molecular patterns (PAMPs) of bacteria make them highly immunogenic, which further improve the specific immune recognition and elimination of cancer cells (4). Indeed, bacteria-based immunotherapy is of great potential to enhance the antitumor effect and is gradually being developed (5).

Bacteria act as natural immune antigens, stimulating the immune system to kill tumors by activating immune cells. In the 19th century, William B. Coley pioneered the use of bacteria for cancer immunotherapy by treating sarcomas patients with the injection of streptococcus pyogenes (6, 7). Further discovery of a growing number of gut bacteria that can be used to fight immune elimination and immune escape in cancer (3, 8). Recent clinical trials show Actinobacteria of Firmicutes and Lachnospiraceae/Rumococcus provoke strong antitumor immunity (9). Further understanding of bacterial-host interaction in the immune environment promotes improved tumor immunotherapy (10). With in-depth research, bacterial antigens, and bacterial outer membrane vesicles (OMVs) (11, 12), have been shaped into favorable immune activators to fight cancer (13). In addition, personalized engineered bacteria have been developed to enhance the antitumor immune response (14, 15). For example, attenuated Listeria-based vaccines effectively activate antitumor immune responses and eliminate tumors (16). There have also been several effective strategies that combine bacterial immunotherapy with conventional therapies (17). However, clinical transition still exists issues, such as objective adverse immune events and biosafety concerns (18).

As mentioned above, naive bacteria and modified bacteria can both activate the antitumor immune response. Here, we focus on reviewing the recent progress on bacteria attenuated techniques, diverse use patterns for bacterial vaccines, and emerging combination therapy. The opportunities and challenges between the preclinical research on bacterial immunotherapy and the need for clinical application are discussed. To further consolidate the knowledge base used to improve immunotherapy for tumors by learning about new preclinical findings in cancer immunotherapy.

## Bacteria-based natural antitumor immunity roles

**Figure 1:**
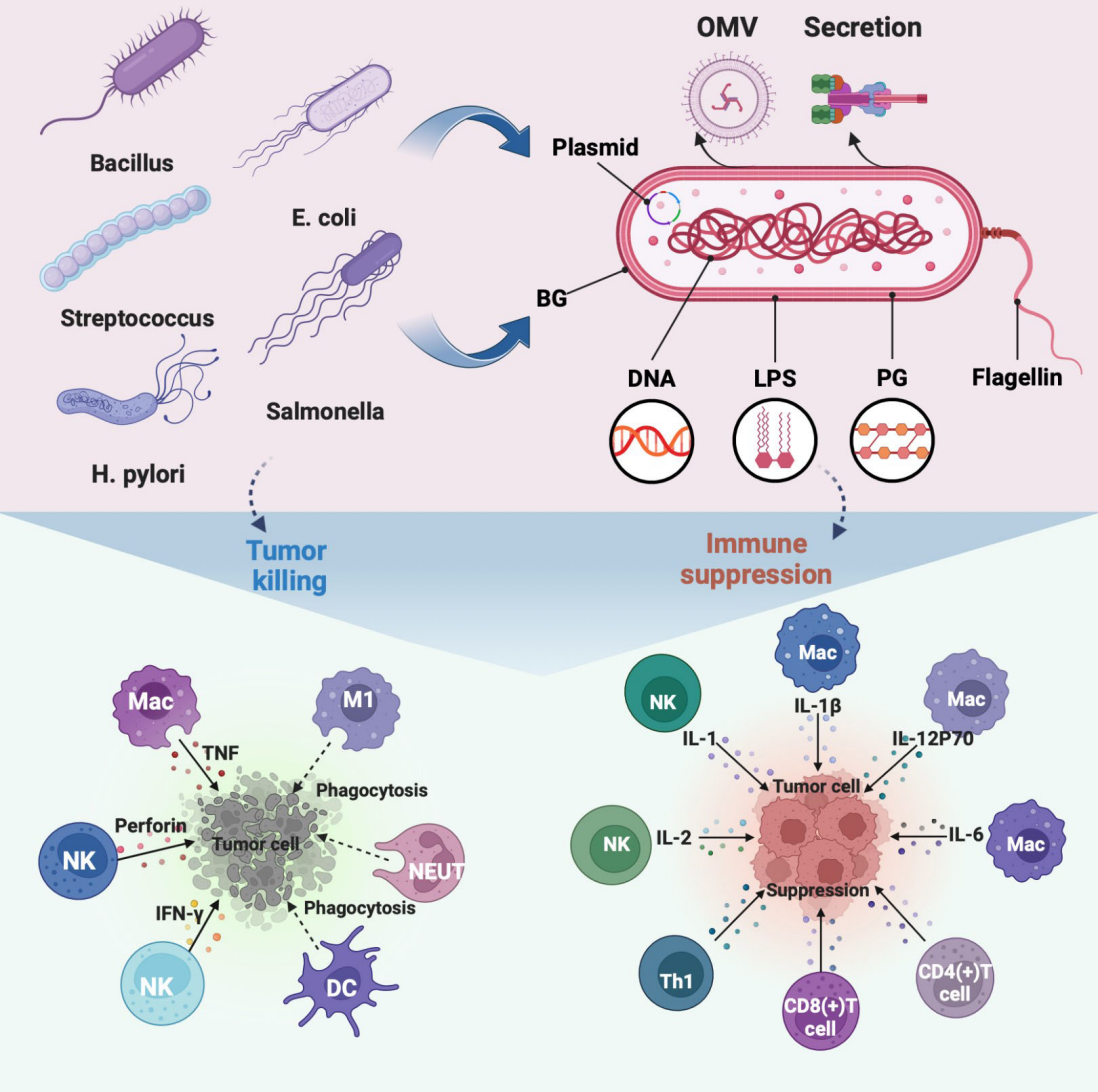
Regulation of immune cells in TEM by representative bacteria, bacterial components, and bacterial derivatives, including the direct killing of tumor cells and suppression of the immunosuppressive environment by activating immune cells to release immune factors. The illustration is drawn on the BioRender website.

Numerous studies have been conducted on the intricate immune response of bacteria in the tumor environment (14, 19, 20). In several cancer models, it has been discovered that bacteria specifically colonize tumors, multiply, have tumor-lysing effects, and stimulate the immune system (21, 22). In addition, bacteria, components, and related derivatives contain a variety of pathogen-associated molecular patterns that trigger immune responses (23, 24) (**Figure 1**). The key factor in the effectiveness of bacterial antitumor immunotherapy has been identified through a review and analysis of bacteria-based tumor immunomodulatory effects.

### Naive bacteria

The discovery of tumor regression caused by bacterial infection opened up bacterial direct antitumor immunotherapy (25). The extensively researched agent in bacterial-mediated cancer therapy is Salmonella, which has a strong potential to induce direct tumor cell death and manipulate the immune elements of the tumor microenvironment (TEM) to assist tumor inhibition (26, 27). For example, tumor-programmed death-ligand 1 (PD-L1) expression can be downregulated by Salmonella, which alters tumor immune tolerance (28). Based on this feature, a recent research study increased the effectiveness of PD-L1 blockers in the treatment of Salmonella colorectal cancer (29). Salmonella may increase the therapeutic benefits of ICIs in the treatment of cancer. Besides, Salmonella is one of the strong candidates to overcome current obstacles to cancer immunotherapy. Three main methods direct antitumor, drug or antigen delivery, and combination immunotherapy can be used by Salmonella to treat cancer (30, 31).

Substantial reported clinical trials show a strong link between intestinal flora and anti-cancer immunity (32–35). Firstly, gut bacteria can act as immune adjuvants to improve the therapeutic coverage and potentially limit ICI toxicity (36, 37), such as the combination of Escherichia coli (E.coli) and TGF-beta blockade galunisertib (Gal) significantly boosts antitumor immunity (38). Probiotics, such as Lactobacillus, Bifidobacterium, Helicobacter pylori (H.pylori), and Lactobacillus rhamnosus probiotic-M9, have come into focus as the driving force behind this synergistic effect (39–41). Recently, a mixture of four Clostridium strains (CC4) has been used to successfully optimize anti-PD-1 therapy (41). By colonizing them separately, it was concluded that they might function as independent immunotherapeutic agents for melanoma and colorectal cancer (CRC) (42). There is hope for more strategies of bacterial synergistic immunotherapy for cancer. As akkermansia muciniphila induces immunoglobulin G1 (IgG1) antibodies and antigen-specific T cell responses in mice, mechanisms by which intestinal bacteria increase immune effects are being studied in greater depth (43). Emerging research validates that uropathogenic E. coli (UPEC) derived from prostatic patients can enhance the immunogenicity of drug-resistant cancer cells and restore immune response (44). Enough to understand the importance of gut microbiota in cancer immunotherapy.

The discovery of more natural bacteria for various immunomodulatory functions is ongoing. For example, colonization of Helicobacter hepaticus (Hhep) in CRC mice boosted tumor infiltration by T follicular helper (Tfh) cells and limited tumor growth (45). Besides, a retrospective clinical trial showed that the manipulation of the gut microbiota by C. butyricum MIYAIRI 588 (CBM588) greatly increased the efficacy of ICI in treating lung cancer (46). However, adverse immune responses remain unaddressed due to bacterial toxicity and individual variability (47). With the advancement of bioengineering technology, one trial evaluated transferrin-modified nano-thin polyelectrolyte shell-coated Subtilis cells *in vitro*, which induces leukemia cell death more strongly than controls (48). The modification mode weakens the immunogenicity of the host to bacteria while optimizing the targeting ability of bacteria in the complex TEM (47).

### Bacterial components

Not only bacteria but also bacterial components behave as immune adjuvants. Recent research reviewed the essential mechanism by which bacterial flagellin acts as an immunomodulator (49, 50). On the one hand, it reveals that flagellin recognizes receptors specifically with the germ line-encoded pattern. Highlighting the physical connection of flagellin to antigens enhances the activation of immune cells. On the other hand, the interaction of flagellin with highly immunogenic tumors induces Th1 responses and suppresses Tregs, thereby inhibiting tumor growth independently (51). It is further convincing that Salmonella typhimurium (S. typhimurium) was modified to express flagellin B, which enhanced tumor immune control *via* a TLR-dependent mechanism (52).

The immunogenicity of bacterial wall components is well-used in antitumor therapy (53). For example, monophosphorylate lipid A (MPLA) was designed to eradicate toxic proinflammatory effects while maintaining sufficient immunogenicity (54). Emerging data show that beta-glucan and muramyl peptides from bacterial walls specifically recognize receptors to stimulate immune cells (55, 56). Besides, lipopolysaccharide (LPS) increased the effectiveness of adoptive CD8(+) T-cell transfer in CRC patients while weakening tumor suppressive immunity (57, 58). Additionally demonstrated to be outstanding immunomodulators in the TEM, modified LPS on bacteria offer significant potential for immunotherapy (59). Interestingly, bacterial ghosts (BGs) were found to be substantially more immunostimulatory than LPS (60). BGs are empty envelopes of gram-negative bacteria, which can effectively induce immune cells to produce long-term antitumor immune memory effects (61). Bacterial cyclic dinucleotides (CDNs) were also found to amplify immune effects in tumor regions by triggering the STING pathway (62). Overall, bacterial components have the potential to be used as anticancer immune adjuvants in therapeutic settings. Further needs to be combined with continually improving medical technology to develop into a mature therapeutic agent.

### Bacterial derivatives

More naturally occurring active compounds released by bacteria have been identified as a result of contemporary bioprospecting approaches (63). The beneficial immune regulation of bacterial metabolites in the oncogenic mechanism is becoming clear (64, 65). Monophosphorylate lipid A (MPL) was the first bacterial immune adjuvant approved for human use. But it does insufficiently elicit an effective response in weakly immunogenic tumors (66). There is considerable scientific evidence that bacterial toxins can activate antitumor adaptive immune as immunotherapeutic proteins (67). However, when colonized in patients, their innate immunogenicity renders them inert against malignancies. In addition, there is an antitumor mechanism of bacterial metabolites based on immunotherapy activation. Under the premise that T cells have been stimulated with ICIs to express adenosine A2A receptors, B. pseudolongum-derived inosine could enhance antitumor immunity by co-stimulation (68). Some bacterial secretions allow for indirect manipulation of the immune system in malignancies. For example, the transforming growth factor-β1 (TGF-β1) produced by certain strains promotes the secretion of interferon-γ (IFN-γ) to inhibit B16F10 tumor growth (69). Taken together, bacterial metabolites have the potential to be developed as antitumor immunotherapeutic.

Bacterial membrane vesicles (BMVs) gain widespread attention in the biomedical field, such as bacterial outer membrane vesicles (OMVs), inner membrane vesicles (IMVs), and double-membrane vesicles (DMVs) (70). BMV function as adjuvants by increasing antigen absorption and presentation to activate innate immunity (71). Further have found their ability to specifically target tumors, BMVs may be used as a drug delivery vehicle to increase the accumulation at the tumor site (72, 73). For example, doxorubicin-loaded with OMVs or IMVs, can be transported more efficiently to non-small lung cancer A549 cells (74, 75). More importantly, pathological antigens, PAMPs, and other immunostimulatory molecules are contained in BMVs (76), which is beneficial for the development of bacterial vaccines (77). For example, vaccinations based on P. aeruginosa-derived DMV successfully induce innate and adaptive immune responses in mouse models (78). In addition, the nano size of BMVs facilitates accumulation in tumor regions, further enhancing the permeation and retention (EPR) effect to trigger an antitumor immune response. Because of these qualities, BMV is expected to participate in antitumor treatment as an immunotherapeutic agent. For example, OMV of similar size to diseased antigens has been designed as an effective antitumor immune vaccine (79). Interestingly, a study artificially produced a non-toxic synthetic bacterial vesicle (SyBV). SyBV, when combined with other immunotherapeutic drugs, activates immune cells, producing melanoma regression, and better adjuvant effects increasing anti-PD-1 inhibitor therapeutic efficacy (80). Furthermore, bacteria-derived vesicles can be genetically and chemically manipulated to serve as vaccines or vaccine vectors, making them a promising cancer treatment option (73).

## Effects of bacterial therapeutic agents on immune cells

### Regulating nonspecific immune cells

The non-specific immune function of phagocytes is the first line of defense against pathogenic invasion of the immune system. Non-pathogenic bacteria can aggressively target phagocytes even though they cannot actively enter host cells. This characteristic was used to create transgenic bacteria that target tumors and promote the “passive transfection” of phagocytes (81). Typical phagocytes that modulate the tumor immune microenvironment include macrophages, dendritic cells, and neutrophils. (**Figure 1**) Some engineered bacterial therapeutics, such as Salmonella coated with allylamine hydrochloride and engineered FlaB-secreting bacteria, are colonized in TEM to promote the infiltration of numerous macrophages and neutrophils (49, 82). Specifically, high levels of macrophage maturation factors were detected in NSI mice injected with live BCG, such as IL-6, IL-1β, IL-12P70, TNF superfamily member 11, tumor necrosis factor-α and monocyte chemotactic protein 1 (83). Besides, specific nanomaterials are selected to modify bacteria into nanocomposites, such as PLGA-R848, and polydopamine binds to the surface of E. coli, which can induce or promote the polarization of macrophages from M2 type to pro-inflammatory M1 type (84, 85). Similarly, Akkermansia muciniphila-derived OMV polarizes macrophages as one of the immune mechanisms against pancreatic tumors (86).

Early induction of monocyte-derived dendritic cells is one of the indications of successful cancer immune response activation (87). Factors derived from bacteria are often used as immune adjuvants for DC-based antitumor therapy, such as the 50S ribosomal protein and w-chorismate mutase TBCM of Mycobacterium tuberculosis, FcγR secreted by Staphylococcus aureus (87–89). TBCM activates DC in a dose-dependent manner through TLR4-mediated signaling, including upregulating co-stimulatory molecules, increasing pro-inflammatory cytokine secretion, and migration, and triggering Th1 immune response (88). Besides, cross-presentation of DCs within the tumor region can also be initiated by direct colonization of Bifidobacteria *via* the STING pathway (90). Moreover, the co-delivery of bacteria with other adjuvants aids in DC maturation (91). For example, the co-delivery of cysteine protease inhibitor U-Omp19 with Ag of Brucella can lengthen the half-life of Ag in DCs, which aids in the sustained expression of MHC class I/peptide complexes on the DC surface (92). As a result, when U-Omp19 increases Ag cross-presentation from DCs to CD8(+) T cells, a powerful antitumor adaptive immune response is triggered (92).

Natural killer cells (NKs) are members of the innate lymphocyte (ILC) family, which exhibit pleiotropic immune behavior in tumors (92). Bacteria, such as Salmonella and Mycobacterium, can inhibit the spread of many cancers in an NK cell-dependent way (93, 94). Mechanistically, NK cells are stimulated by bacteria to produce IFN-γ, which regulates their accumulation, activation, and cytotoxicity to inhibit tumor metastasis (95). More comprehensively, more cytokines, including TNF-γ, perforin, IL-2, and IL-1, were validated in a preclinical study in which the Nocardia redis cell wall skeleton (Nr-CWS) inhibited melanoma-induced lung metastasis in mice (96). Besides, it was found that the expression of CD69, TRAIL, and FasL on NK cells increased, leading to the hypothesis that one of the anti-metastatic mechanisms is promoting the terminal differentiation of NK cells (96). Bacteria-derived immunogenic substances, such as Clostridium clostridia-spores (CVN-NT) and the E. coli fusion protein dsNKG2D-IL-15, can also support the continuation of NK cell toxic function on tumor cells (97, 98).

### Regulating specific immune cells

Numerous preclinical evaluations have shown that bacteria rely on T cells to generate antitumor adaptive immune responses (99) (**Figure 1**), such as the CD3(+) T cell immune response activated by the Helicobacter pylori vaccine to inhibit GC cell growth (100). Through the analysis of Listeria, E. coli in common immune models, it was revealed that bacterial immune antigens activated CD8(+) T cells in the induction phase to eliminate tumors, and simultaneously promoted CD4(+) and CD8(+) T cells to eradicate tumors (19, 101, 102). Further found that bacteria primarily induce specific CD4(+) or CD8(+) T cell immune function enhancement through IFN-signaling in tumors, such as attenuated Listeria monocytogenes (LADD-Ag) and BCG (103, 104). Besides, adoptive metastasis specific to Bacteroides fragilis T cells can address the ineffectiveness of CTLA-4 blockade, which occurs in antibiotic-treated conditions or a sterile tumor setting (105). Interestingly, SVYRYYGL (SVY) immunity derived from the commensal bacterium Bifidobacterium breve, which refers to T cells targeting an epitope, such as SVY-specific T cells cross-reacting with the melanoma antigen SIYRYYGL (106). Furthermore, bacteria can be engineered to enhance tumor-infiltrating T cells by regulating chemokines or directly upregulating L-arginine concentration (107, 108), such as S.typhimurium A1-R and E.coli expressing CD47nb (109, 110). Overall, bacterial therapeutics have the potential to induce or enhance the immune response of T cells in favor of tumor eradication.

## Bacteria-based preclinical treatment modes

**Figure 2:**
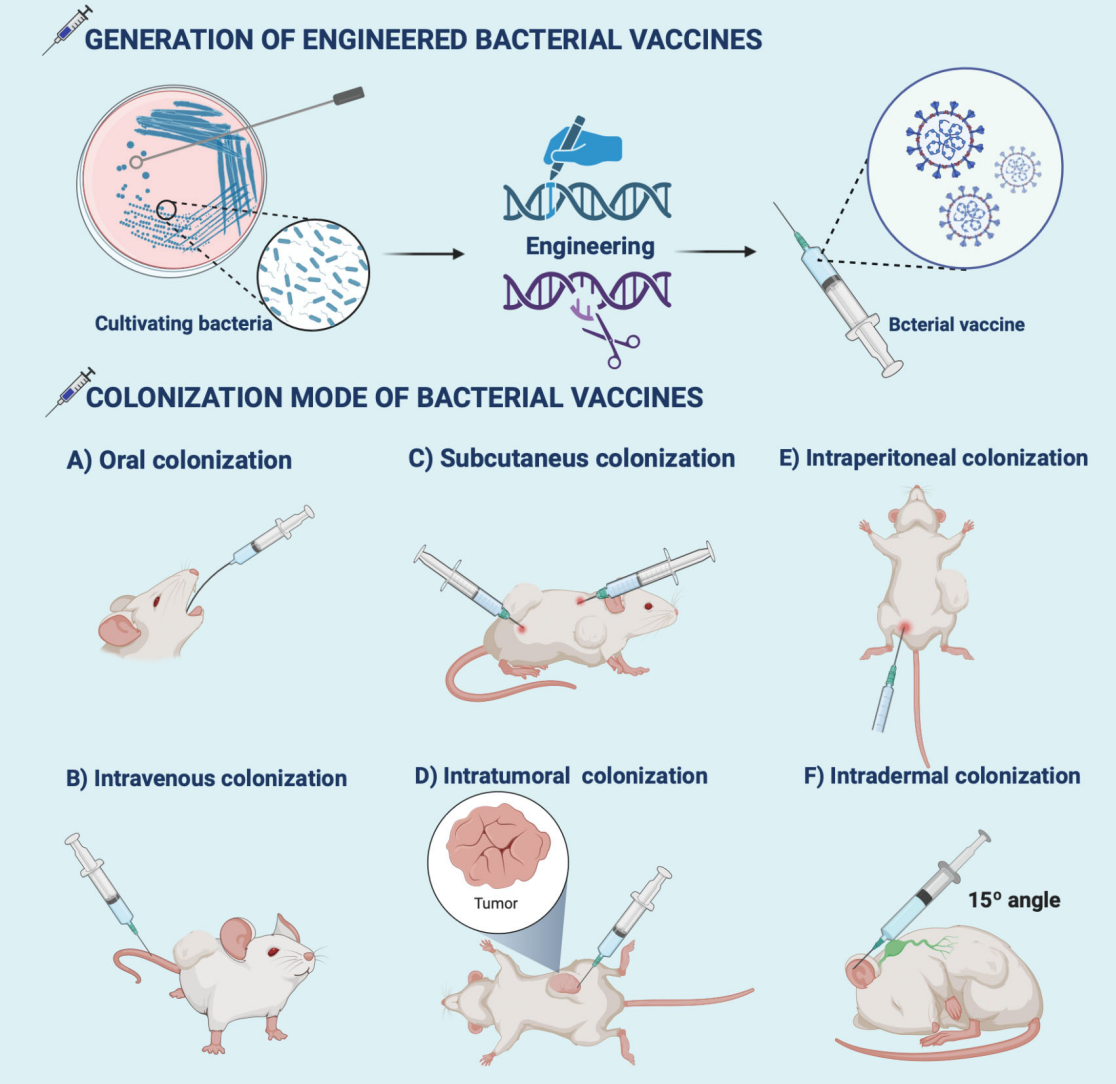
Generation and colonization modes of bacterial vaccines. Bioengineering techniques such as genetic engineering facilitate the generation of bacterial vaccines. The commonly used colonization modes of bacterial vaccines developed in preclinical antitumor research include **(A)** oral, **(B)** intravenous, **(C)** intratumor, **(D)** subcutaneous, **(E)** intraperitoneal, and **(F)** intradermal injections. The illustration is drawn on the BioRender website.

Routine colonization of bacteria-based vaccine formulations in oncology is still limited by biological barriers (111). For example, Clostridium endophytic spores are poorly immunogenic, do not induce an immune response, and remain in multiple normal organs after intravenous injection, but reach the tumor region triggering a strong inflammatory and immune response that massively destroys the tumor (112). So, it is inferred that intratumoral injection may better reflect the medical application value of the antigen. Special emphasis is given to rational colonization of bacterial formulations which is beneficial for the immune response to penetrate the TEM (113) (**Figure 2** and **Table 1**). We also reveal that practical animal models are the key to the clinical success of immunotherapy (155, 156). Provide a reference for creating potential opportunities for clinical translation of bacterial vaccinology research.

**Table 1:** Colonization modes of bacterial vaccines for antitumor immunotherapy.

Colonization mode	Vaccine composition	Tumor model	Immunotherapy role	Refer
Oral	Attenuated Salmonella carrying encoding VEGFR-2	Advanced pancreatic cancer	Increasing VEGFR2-specific Teff	(114)
	Salmonella Typhi vaccine strain CVD 915	Liver metastases tumor model	Inhibiting liver metastases	(115)
	Live attenuated Salmonella coated with cationic polymers and plasmid DNA	B16F10 melanoma model	Showing remarkable T cell activation and cytokine production	(116)
	Attenuated S. typhimurium strain SL7207	MYCN-negative mammary carcinoma model	Mediating immune response	(117)
	Bifidobacterium longum displaying a partial mouse Wilms’ tumor 1 protein	Castration-resistant prostate syngeneic tumor model	Inhibited tumor growth	(118)
	Bifidobacterium longum displaying a partial mouse Wilms’ tumor 1 protein	C1498-WT1 murine leukemia syngeneic tumor model	Inducing WT1-specific cellular immunity	(119, 120)
	Recombinant lactococci expressing inducible E6 oncoprotein	TC-1 tumor model	Inducing humoral and cellular immunity	(121)
Intravenous injection	E. coli-plant hybrid vesicles	CT26 colon tumor model and Luc-4T1 breast tumor model	Stimulating the activation of immune cells	(122)
	Red blood cell membranes encapsulated Lmo with selective deletion of virulence factors	CT26 colon tumor model	Reversing immunosuppressive TEM and promoting systemic strong and durable antitumor immune response	(123)
	Attenuated S. Typhimurium strain, BCT2	Engineered breast cancer mouse model	Eliminating toxic immune response signals against bacteria	(124)
	Spores of genetically engineered Clostridium	CT26 colon tumor model	Stimulating proliferation of T cells	(125)
	E. coli MG1655 expressing INF-γ	4T1 breast tumor model	Activating the antitumor immune response	(126)
	BCG cell wall skeleton and ovalbumin-loaded NP	E.G7-OVA lymphoma model	Resulting in the generation of ova-specific cytotoxic T cells and inhibiting the tumor growth	(127)
	Attenuated Salmonella derived-OMV-coated polymeric micelles	B16F10 melanoma model	Killing cancer cells directly	(128)
	SL7207: an auxotrophic Salmonella enterica serovar Typhimurium aroA mutant	B16F10 melanoma model	Infiltrating inflammatory monocytes for tumor growth inhibition	(129)
	Doxorubicin loaded in E. coli-derived nanovesicles	B16F10 melanoma model	Enhancing the delivery of therapeutics to TEM	(130)
	Aptamer-conjugated attenuated Salmonella	4T1 and H22 tumor-bearing mouse models	Enhancing antitumor efficacy	(131)
	The lipid-coated EcN	4T1 breast tumor model	Increasing their biocompatibility with blood cells and the immune system	(132)
	Brucella melitensis 16M ΔvjbR, henceforth BmΔvjbR	A murine colon adenocarcinoma model	Promoting macrophage and T cell-mediated antitumor immunity.	(133)
Intratumoral injection	Engineered E. coli SYNB1891	B16F10 melanoma, EL4 lymphoma, A20 lymphoma, 4T1 breast tumor model	Activating complementary innate immune pathways	(134)
	Engineered E. coli NISSLE1917	*In situ* colon and breast tumor model	Temporarily evading immune attacks and improving antitumor efficacy	(135)
	Auxotrophic Salmonella vector strain SF200	CT26 colorectal tumor model	Increasing immune-stimulatory capacity and overcoming the efficacy-limiting effects of pre-exposure	(136)
	E. coli vehicle engineered for a high secretion level	B16F10 melanoma model	Remodeling the TEM in favor of several antitumor immune cells	(137)
	Zn-, and Mg-containing tricalcium phosphates loaded with a hydrothermal extract of a human tubercle bacillus	Lewis lung carcinoma (LLC) model	Eliciting potent systemic antitumor immunity *in vivo*	(138)
	A probiotic food-grade Lactococcus lactis	CT26 colon tumor model	Converting the “cold” tumors to “hot” tumors	(139)
Intraperitoneal injection	Vibrio vulnificus FlaB with HPV 16 E6/E7 short peptide	TC-1 tumor mode	Inducing long-term antitumor immune responses.	(140)
	Recombinant Nap protein (HP-Nap) from Helicobacter pylori loaded into synthetic chitosan	4T1 breast tumor model	Regulating cytokine production and activating the immune system to kill tumor cells	(141)
	Listeria expressing the tumor-associated antigen Mage-b	4T1 breast tumor model	Increasing activity of NK cell and T cell responses	(142)
	A Listeria (Lm) encoding an antigenic fragment of CD105 (Lm-LLO-CD105A)	Subcutaneous and orthotopic renal cell carcinoma models	Resulting in increased infiltration of polyfunctional CD8(+) and CD(4+) T cells and reducing infiltration of immunosuppressive cell types	(143)
Subcutaneous injection	A demi-bacterium from Bacillus	EG.7‐tumor model and 4T1 breast tumor model	Achieving synergistic induction of immune responses	(144)
	Engineered E. coli encapsulated by Chitosan microspheres	B16-OVA melanoma model	Activating specific immunity and achieving tumor prevention	(145)
	Mycobacterial outer membrane attached tumor-specific peptides	B16F10 melanoma model and colorectal tumor model	Inducing strong systemic and intratumoral T cell-specific immune responses	(146)
	Bioengineered E. Coli-derived OMVs loaded with different tumor antigens	B16F10, B16-OVA melanoma model, MC38 colorectal tumor model, Pan 02 pancreatic tumor model	Eliciting a synergistic antitumor Immune response	(147)
	Recombinant OMV derived from the recombinant plasmid-transformed BL21 (DE3) Escherichia coli	B16-OVA metastatic tumor model and MC38 colorectal tumor model	Promoting cross-presentation of DC cells	(148)
	Bacterial cytoplasmic membrane from E. coli and tumor cell membrane from tumor tissues coated onto the PLGA nanoparticles	B16F10, B16-OVA melanoma model, MC38 colorectal tumor model, CT26 colon tumor model	Inducing effective antigen presentation and robust adaptive immune activation	(148)
	Recombinant Salmonella containing CEACAM6 and 4-1BBL	DMH colorectal tumor model	Enhancing T-cell immunity and inhibiting the development of colorectal cancer	(149)
	Glutaraldehyde-fixed HUVEC conjugated with 2 repeats of mycobacterial HSP70 (407–426) (M2)	H22 hepatocellular carcinoma model	Enhancing antitumor efficacy	(150)
	Attenuated S. typhimurium expressing recombinant IFN-γ	B16F10 melanoma model	Inhibiting tumor growth in an NK cell-dependent manner	(151)
	A nanoparticulated conjugate of heat-stable enterotoxin of enterotoxigenic E. coli	A549 lung tumor model	Inducing immune response and neutralizing antibody Titer	(152)
	The type III secretion system of attenuation P. aeruginosa	B16-OVA melanoma model	Inducing a cellular immune response to the cancerous cells	(153)
Intradermal injection	Bordetella pertussis	Cervical tumor model	Inducing Th1-polarized CD8(+) cytotoxic T-lymphocyte responses	(154)

### Oral bacterial vaccine

Oral tumor vaccines have been shown to successfully cross the intestinal epithelial barrier, be taken up by DCs in the intestinal lamina propria, result in the production of draining lymph nodes and the presentation of tumor antigens, and ultimately induce a potent antitumor immune response (157). For example, autologous vascular endothelial growth factor receptor 2 (VEGFR2) was successfully delivered into tumor blood vessels by an attenuated Salmonella-based oral-engineered vaccine, and tumor growth in B16F10 mice was subsequently inhibited by significant T cell activation (116). In a similar experiment, the Salmonella Typhi vaccine strain CVD915 was given orally to breast cancer mice to assess its effectiveness against tumors and ability to prevent liver metastasis (115). Such heartening preclinical outcomes supported the creation of the first oral cancer vaccine, VXM01, whose antitumor immune reaction was evaluated and authorized in patients with prostate cancer (115). More well-established preclinical studies explored oral doses suitable for humans. For example, the recombinant Bifidobacterium longum oral vaccine is precisely quantified for the treatment of castration-resistant prostate cancer (CRPC) (118). The rich treatment mode has broadened the range of applications for bacterial vaccines in the challenging clinical tumor treatment environment.

### Intravenous bacterial vaccine

Due to their massive dosages, quick medication effects, and minimal toxicity and side effects, intravenous vaccinations have been widely used for the treatment of cancer. For example, cancer vaccines based on bacterial-plant hybrid vesicles (BPNs) were injected intravenously into CT26 and Luc-4T1 tumor models to study the immunotherapy effect (122). This intravenous vaccine still has some limitations in terms of clinical use, such as rapid clearance and increased bacterial toxicity due to systemic administration. However, there is mounting proof from considerable preclinical studies that intravenous bacterial vaccines have an antitumor effect. For example, based on genomic detection of bone tumors after intravenous red blood cell membranes encapsulated Lmo (Lmo@RBC), researchers found that the therapeutic agent activated cell pyroptosis to reverse the immunosuppressive TEM and enhance systemic antitumor immunity (123). The discovery of emerging mechanisms also has lain a theoretical foundation for the upcoming creation of multifaceted bacterial vaccines. Preclinical studies using transplanted tumor models in mice and clinical trials using human patients produce different results. A genetically modified S.typhimurium can be intravenously colonized into mice and humans with breast cancer to the same extent to overcome this significant limiting factor in clinical translation (124). Besides, preclinical experimental research is now being conducted on numerous intravenous bacterial agents. The main innovative parts are immunotherapy systems based on engineered bacteria and OMVs, such as spatiotemporal control of engineered bacteria and self-Blockade of PD-L1 with bacteria-derived OMVs (126, 158). Their excellent immune antitumor effects have been demonstrated in a variety of tumor models, such as melanoma, 4T1 tumor, and colon tumor models. The blind zone in the development of antitumor bacterial vaccines is supplemented and optimized from all directions.

### Intratumoral injection of bacterial vaccine

Cancer immunotherapy can be realized by directly injecting bacterial therapeutic agents into the tumor site, which has the advantage of lower tolerability and higher safety over intravenous injection. Preclinical studies with significant antitumor effects are now available for reference. For example, engineered E. coli SYNB1891 was colonized *in situ* into the four tumors, including melanoma, EL4 lymphoma, A20 lymphoma, and 4T1 breast tumor. It was revealed in detail that this intratumoral therapeutic agent can activate complementary innate immune pathways by stimulating the STING channel (134). This kind of work aims to achieve antitumor immunity while building biocontainment capabilities to meet producibility and regulatory standards (134). Besides, glycoproteins can be recognized by the immune system to stimulate innate and humoral immunity (159). Novel vaccines using polysaccharide-encapsulated bacteria can exert a key inhibitory effect on malignant tumors (159). However, several clinical trials have fallen short due to related immune tolerance. More advanced, E. coli NISSLE1917 is programmed to regulate the dynamic expression of surface capsular polysaccharides, resulting in high immune tolerance *in situ* colon and breast tumor encapsulation (135). The novel finding sheds new light on the tolerable dose of novel antitumor bacterial vaccines (136). Overall, it paves the way for the development of a multimodal, versatile bacterial vaccine.

### Intraperitoneal injection of bacterial vaccine

Because of the large surface area of the peritoneum, intraperitoneal injection absorbs quickly, is easy to use, and offers a wide range of uses. The use of this colonization model for novel antigens is expected to further overcome the limitations of tumor immunosuppressive microenvironment. For example, a flagellin-adjuvanted HPV E7 vaccine was engineered to express the long peptide E7-LP35 which shows excellent immunogenicity (140). It was injected intraperitoneally into a mouse TC-1 tumor model to enhance the effective immune response (140). Besides, it is possible to isolate immunological components from bacteria to obtain effective vaccination antigens. For example, recombinant Nap protein (HP-Nap) derived from Helicobacter pylori was loaded into synthetic chitosan to form nanoparticle complexes, which were injected intraperitoneally into 4T1 mice to regulate cytokine production and activate the immune system to kill tumor cells (141). For the study focused on breast cancer metastasis,a vaccine developed based on Listeria expressing the tumor-associated antigen Mage-b, which effectively inhibited tumor metastasis but left serious hepatotoxicity (142). The newly designed galactosyl ceramide complex of Listeria almost abolished cancer cell metastasis without concomitant toxicity (142). In general, recombinant antigens with different modifications can be colonized intraperitoneally to achieve cancer targeting with bacterial vaccines.

### Subcutaneous injection of bacterial vaccine

The therapeutic effect can be prolonged by using subcutaneous injection absorption, which is slower than intravascular absorption but faster and more precise than oral and enema methods. Numerous studies have found that using less risky subcutaneous injections rather than intravascular ones, tailored bacterial nanovaccines can reduce the intrinsic immunogenicity of bacteria. For example, a biomimetic vaccine based on a demi-bacteria (DB) from Bacillus achieves synergistic induction of cellular and humoral immune responses in a subcutaneous injection mode (144). Besides, OMVs are considered suitable vaccine candidates with their unique immunity (77, 147). Recently some powerful OMV-based vaccines have been subcutaneously colonized into different cancer models. For example, the OMV-LL-mRNA vaccine can protect mice by inducing a long-lasting adaptive immune response while also quickly inhibiting the growth and metastasis of colon and melanin tumors (148). More advanced, a self-assembled protein nano vaccine (BMC-OVA) has been formed by fusion in hydrogel-coated engineered E. coli (145). After the engineered bacteria microcapsule was subcutaneously colonized into the mouse breast tumor model, the active release of BMC-OVA triggered specific immunity to lyse tumor cells B16-OVA (145). These are promising strategies to produce clinic subcutaneous long-acting immune vaccines.

### Intradermal injection of bacterial vaccine

Bacteria have been extensively used in the creation of active immune vaccines for the treatment of cancer. The long-established antitumor vaccine Bacille Calmette-Guerin (BCG) is usually administered intradermally to prevent and treat noninvasive bladder cancer (160, 161), and is known for high efficiency in small doses. Other newly developed recombinant BCG (rBCG) has been subcutaneously colonized in mouse models. They have reduced toxicity and enhanced the expression of tumor-associated antigens (TAAs) (162–164). Additionally, several bacterial cancer vaccines are presently undergoing clinical development (165). For example, clinical trials of recombinant CyaA vaccines for intradermal injection cervical cancer treatment (166). CyaA is a vital virulence protein of Bordetella pertussis that suppresses the adaptive immune response by regulating dendritic cells. Certain recombinant CyaA toxins have been used as vaccination carriers to spread the HPV virus and cause Th1-polarized CD8(+) cytotoxic T-lymphocyte responses (154). The effectiveness of the pertussis vaccine in mice can also be increased by co-injecting CyaA with pertussis bacillus anti-donation. Further expected that the number of powerful bacterial vaccines could be expanded in the future.

## Development of bacteria-based combination treatment

**Figure 3:**
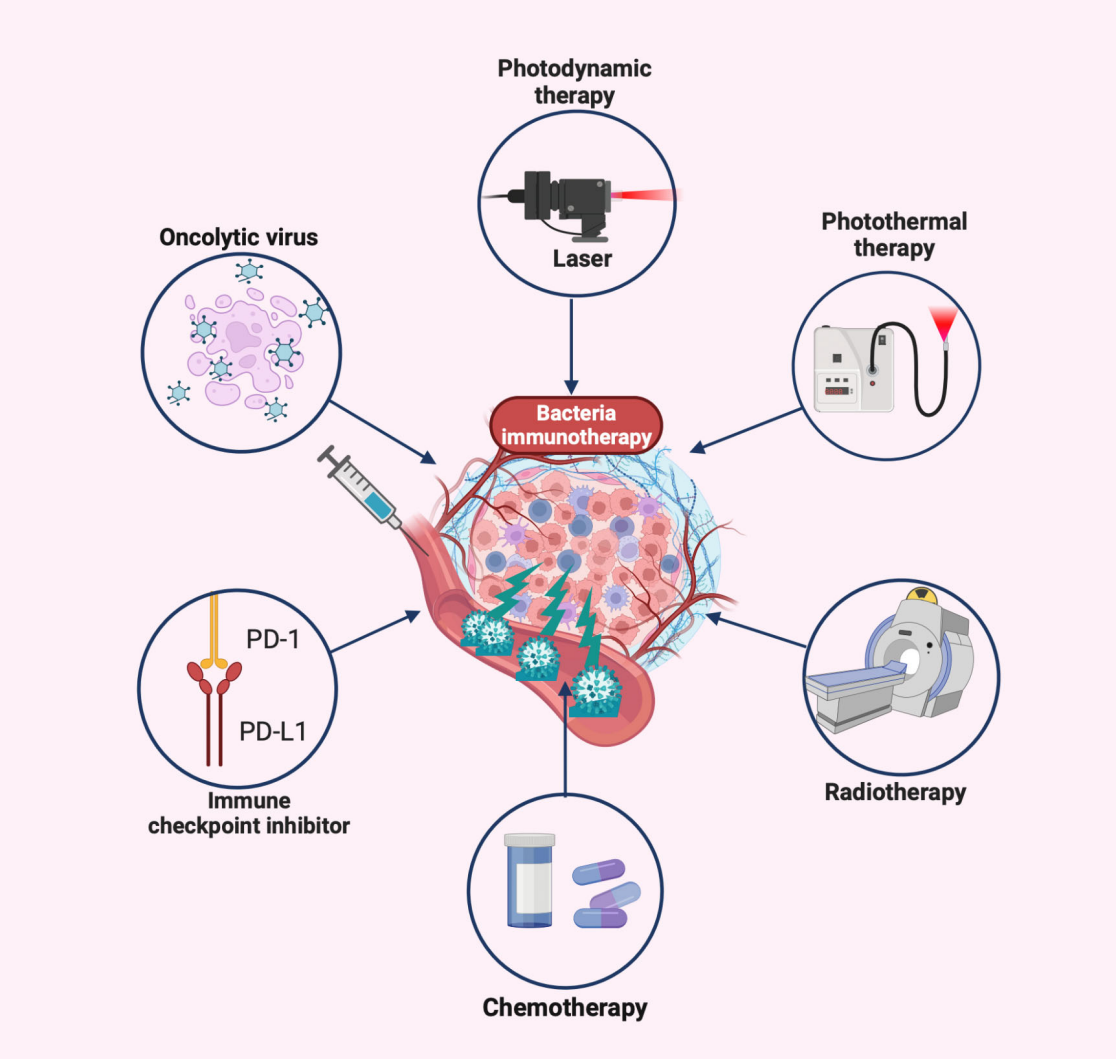
Bacteria-based combination antitumor immunotherapy. Bacteria immunotherapy in combination with other therapies promotes antitumor treatment, generally including photodynamic therapy, photothermal therapy, radiation therapy, chemotherapy, immune checkpoint inhibitors, and oncolytic viruses. The illustration is drawn on the BioRender website.

The inability to deeply eradicate tumors and adverse side effects are caused by the toxicity of traditional cancer treatment to normal cells and the tolerance of tumor cells (167). Some of the drawbacks of conventional therapy have been effectively addressed by the creation of novel bacterial antitumor immunotherapies by the development of novel bacterial antitumor immunotherapies. Even more intriguing is the preclinical study on various combination immunotherapies for cancer (**Figure 3** and **Table 2**). Bacterial immunotherapy and other single antitumor therapies make up for each other’s deficiencies (22, 207), such as chemotherapy, photodynamic therapy, photothermal therapy, immune checkpoint inhibitor therapy, and oncolytic virus therapy. The bioavailability of various combination therapies is enhanced by the distinctive compatibility of engineered bacteria (5, 208, 209). The tumor-targeting and immune response properties of bacteria are also significantly improved by combined therapeutic techniques. By exploring the innovative and optimal capabilities of these versatile tumor immunotherapy systems, researchers are attempting to surmount the numerous challenges of clinical translation.

**Table 2:** Bacteria-based combination antitumor immunotherapy.

Combined therapy	Combined object	Bacterial therapeutic agent	Tumor model	Refer
PDT	Photosensitizer MG-2I, a LED light (660 nm, 6 mW/cm2)	Engineered avirulent Salmonella expressing a fluorogen-activating protein (FAP)	Subcutaneous HCT116 and CT26 colon tumor models	(168)
	Photosensitizer TPApy, light irradiation (100 mW cm−2)	Clostridium butyrate	B16F10 Melanoma model	(169)
	Photosensitizer CAT-Ce6, laser irradiation (0.15 W/cm2, 10 min)	PD-L1 antibody modified-attenuated Salmonella outer membrane vesicles (OMV-aPDL1)	4T1 breast tumor model	(170)
	Photosensitizer ZnPc, laser (808 nm, MDL-3)	pDawn-IFN-γ engineered L. lactis	B16F10 melanoma model	(171)
	Photosensitizer chlorin e6 (Ce6), D-luciferin	Attenuated S. typhimurium strain ΔppGpp	CT26 colon tumor and VX2 rabbit tumor model	(17)
	Photosensitizer MA, light irradiation (30 mW cm^2^)	Attenuated Salmonella	4T1 breast tumor model	(172)
PTT	NIR	Natural active photosynthetic bacteria (PSB)	B16F10 Melanoma model	(173)
	NIR	Engineered E. coli	CT26 colon tumor model	(174)
	NIR	Telurium nanorods synthesized based on E. coli	4T1 breast tumor model	(175)
	NIR	Rojan bacteria equipped with photosensitive ICG silicon-nanoparticles	GBM-bearing mice model	(176)
	NIR	An artificial microrobot MGBM	Metastatic triple-negative breast tumor model	(177)
	NIR	Attenuated Salmonella:△ppGpp S. typhimurium	CT26 colon tumor,4T1 breast tumor, human malignant glioblastoma U87MG tumor, human pancreatic cancer SW1990 tumors, human lung cancer A549 tumors, human cervical tumor model	(178)
	NIR	TLR-5 adjuvant Vibrio vulnificus FlaB conjugated onto the surface to an IR 780-loaded hyaluronic acid-stearyl amine micelles	TC-1 tumor model	(179)
	NIR	Melanoma cytomembrane vesicles and attenuated Salmonella OMVs	B16F10 Melanoma model, 4T1 breast tumor model	(180)
	NIR	Facultative anaerobic Salmonella VNP20009 with synthesized heptamethine cyanine dyes NHS-N782 and JQ-1 derivatives	B16F10 Melanoma model	(181)
	NIR	OMVs derived from attenuated S. typhimurium	CT26 colon tumor model	(182)
	NIR	Escherichia coli Nissle 1917 (EcN)	CT26 and MC38 colon tumor model	(85)
Radiotherapy	54.0 Gy intensity modulated radiation	A live attenuated Lm bacterium ADXS11-0011 Lm-LLO	Locally advanced anal tumor model	(183)
	Radionuclides (125I/131I)	Micrococcus luteus (ML) and Staphylococcus aureus (SA), and Gram-negative bacteria, including Escherichia coli and attenuated S. typhimurium (VNP)	4T1 breast tumor, CT26 colon tumor, Luciferase-CT26 tumor model	(184)
	Irradiation (5Gy)	Attenuated Salmonella VNP20009 coated with antigen-adsorbing cationic polymer nanoparticles	4T1, CT26 xenograft tumor models	(185)
Chemotherapy	5-fluorouracil	Specific lactic acid bacteria	4T1 breast tumor model	(186)
	Interferon IFN-γ	Attenuated Salmonella	CT26 colon tumor model	(187)
	Doxorubicin/idarubicin	Bacterial protoplast-derived nanovesicles	A549 human lung carcinoma model	(75)
	Natural ursolic acid UA	Attenuated S. typhimurium ΔppGpp/Lux) with nano assemblies	CT26 colon tumor model	(188)
	Au@Pt nanozyme	E. coli	B16F10 melanoma model	(189)
	Galunisertib (Gal)	Escherichia coli strain Nissle 1917 (EcN)	4T1 and H22 subcutaneous tumor model	(38)
	CHOP: cyclophosphamide, doxorubicin, vincristine, and prednisone	Salmonella	B-cell non-Hodgkin lymphoma (B-NHL) model	(190)
	Doxorubicin	Attenuated Salmonella	CT26 colon tumor model	(191)
	α-galactosylceramide	Recombinant Lm: Listeria-Mage-b	4T1 breast tumor model	(142)
ICI	PD-L1 mAb	An LyP1 polypeptide-modified E. coli -derived OMV(LOMV)	4T1 breast tumor, CT26 colon tumor and B16F10 melanoma model	(158)
		Attenuated Mycobacterium MTBVAC	Orthotopic model of bladder cancer	(192)
		An engineered probiotic ECN strain	MC38 colon adenocarcinoma model	(108)
		A hyper vesiculating ECN (ΔECHy)	4T1 breast tumor model and MC38 colon adenocarcinoma model	(193)
		Bifidobacterium	MB49 bladder tumor and B16F10 melanoma model	(194)
	Anti-PD-1 mAb	Salmonella Typhi Porins	B16F10 melanoma model	(195)
		E. coli-derived monophosphoryl lipid A (EcML)	B16F10 and B16F10-OVA melanoma model	(196)
		Attenuated S. typhimurium	Human xenograft and murine syngeneic schwannoma models	(197)
		Escherichia coli (E. coli) BL21(DE3) plysS strain to engineer bacteria derived vesicles	B16F10‐luc melanoma model	(198)
		EcN	CT26 and MC38 colon tumor model	(85)
		Lactobacillus kefiranofaciens ZW18, Lactobacillus plantarum MA2, BC299, Lactobacillus acidophilus W563, Bifidobacterium pseudocatenulatum W112	B16F10 melanoma model	(199)
	CTLA-4, PD-L1 mAb	E. coli expressing photothermal melanin	4T1 breast tumor model	(200)
		Ultrasound-controlled ECN	A20 tumor model	(201)
	C5a receptor 1 (C5aR1) inhibitor PMX53 (17)	Antiangiogenic Listeria monocytogenes	TC1, 4T1 breast, EpH4 1424.1 tumor model	(202)
OA	Hydrostomatitis virus (VSV)	E. coli expressing B18R	Lewis Lung Carcinoma and HT29 adenocarcinoma model	(203)
	Oncolytic adenovirus (OA)	Homing tumors E. coli BL21	non-small cell lung tumor model	(204)
	Herpes simplex virus (HSV1716)	Magnetospirillum magneticum AMB-1	Human breast MDA-MB-231, SKBR3, PyMT-TS1, 4T1, EO771 cell line-derived mammary tumor model	(205)
	Oncolytic virus Maraba	Lm	B-cell non-Hodgkin lymphoma model	(206)

### Modulated with photodynamic therapy

The therapeutic effect of bacterial cancer therapy and photodynamic cancer therapy is still limited. Through a clever fusion of these two methods, more innovative cancer immunotherapy is actively sought. Looking back at numerous relevant preclinical studies, the most basic combination is the direct co-delivery of engineered bacteria and photosensitizers to the tumor site. For example, E. coli was designed to express the fluorogenic-activated protein (FAP), and the photosensitizer PS was colonized together in colon cancer models (168). On the one hand, intratumoral bacteria invade the vascular system to form a thrombus, which darkens the tumor site to facilitate NIR absorption. On the other hand, the activation of PS by a specific laser promotes the production of ROS, thereby lysing and releasing antigens to activate immune cells (168). In addition to boosting the immune response triggered by the bacteria against the tumor, the synergistic effect also lessens the harmful side effects of bacteria. Besides, photosensitizers can be designed to load bacteria into biological combinatorial therapeutics, like TPApy was loaded on Clostridium butyrate (169). This wrapped combination prevents the photosensitizer from being destroyed to a certain extent and keeps the photodynamic effect stable.

A better overall antitumor immune effect was demonstrated by PD-L1 antibody-modified attenuated Salmonella vesicles-loaded self-assembled nanocomplexes (CAT-Ce6), which can alleviate tumor hypoxia, promote the aggregation of free Ce6 to enhance the photodynamic killing effect, as well as improve the exposure of tumor-associated antigens to elicit an immune response, eventually, prolong the synergistic effect by prolonged release (170). Another triple combination therapy is that the laser simulates engineered bacteria to produce INF-γ, which directly inhibits tumor growth as a third-step synergistic treatment platform (171). However, using this new class of processualized combination therapies, the efficacy of immunotherapy has only been confirmed in a few tumor models. In one study, an attenuated strain of S. typhimurium was transformed into bioluminescent bacteria by the addition of a plasmid expressing firefly luciferase as an internal light source (17). In melanoma and rabbit tumor models, Luc-S. T (DeltappGpp) together with D-luciferin localization on tumors by hydrogel and continuous luminescence, activating Ce6 to exert significant antitumor immune (17). In comparison to external lasers, bio-spontaneous PDT is a highly efficient and adaptable treatment with no penetration restrictions. Overall, rapid immune response and rapid progression are generally advantages of bacteria-based photodynamic immunotherapy, but the translation to a clinical immunotherapy combo platform remains a challenge.

### Strengthen with photothermal therapy

Photothermal therapy (PTT) is critical for the elimination of advanced malignant tumors. The fundamental process involves increasing NIR absorption near the tumor, which raises warmth and causes tumor ablation (178). Existing antitumor studies have found that the near-infrared chemotaxis of natural bacteria is highly desirable. For example, natural active photosynthetic bacteria (PSB) gather in hypoxic tumor areas with hypoxia targeting, and then strongly absorb NIR to generate heat to kill cancer cells (173). Besides, PSB enhances the immune response of T lymphocytes, thereby realizing synergistic effects of PTT antitumor therapy and bacterial immunity. Its dual efficacy is a key reference idea for bio-intelligent cancer treatment. For example, E. coli was engineered as a heat-inducible bacterium (TIB) expressing PD1, which can both exert photothermal effects and specifically recognize PD-L1 on the surface of tumor cells, thus increasing the role of alleviating the tumor immunosuppressive microenvironment (174). Similarly, E. coli-based tellurium nanorods rely on photothermal characteristics and reprogrammed macrophages to generate the same bacterial immune-PTT synergy (175).

Due to the non-pathogenic nature of EcN and the non-proliferative character of TeNRs, relatively high dosages of Te@EcN can be swiftly metabolized and eliminated in normal tissues (175). These complementary approaches have clinical conversion value and collectively enhance the treatment of individually mediated tumors. Moreover, exogenous photothermal agents are designed to be loaded on bacteria and then co-colonized into the tumor. In an *in situ* glioblastoma mouse model, rojan bacteria equipped with photosensitive ICG silicon nanoparticles were verified to have the synergistic effect of photothermal tumor destruction and immunological antitumor, which was reflected in the prolongation of survival of GBM mice (176). The cleverly designed activated immune response leads to the production of NO, eventually leading to the extension of vasodilation-promoting therapeutics to metastatic triple-negative breast tumors (177). A wide range of prospects for antitumor immunotherapy has been made possible by this self-driving synergistic treatment approach based on bacterial characteristics.

### Improved with radiation therapy

Since the TEM is in a state of immunosuppression, it is difficult for single radiotherapy to release sufficient antigens to activate the antitumor immune response (183, 210). Bacteria could provide distinct benefits as natural carriers for tumor-targeted administration and immune activation (184). Engineering changes enable bacteria to deliver specific tumor antigens that can be employed to boost immune activation during radiation therapy. For example, attenuated Salmonella VNP20009 coated with antigen-adsorbing cationic polymer nanoparticles combined with irradiation, greatly increased the activation of DCs and systemic antitumor effect (185). Moreover, therapeutic radiation can be given endogenously to the tumor site by bacteria, for example, by inactivating microbes with radioactive 125I/131I labeling (184). This ingenious combination allows for a robust and long-lasting internal radioimmune response against malignancies while maintaining a high biosafety profile. The clinical value of bacteria-based immunotherapy with radiation has been further confirmed in anal cancer patients. An engineered live attenuated Listeria monocytogenes (Lm) bacterium(ADXS11-0011 Lm-LLO), is safe in combination with standardized chemoradiotherapy for anal cancer (183). However, the specific therapeutic impact needs to be validated further. Overall, bacteria-based radioimmunotherapy is a promising and transformable combination antitumor strategy (211).

### Combined with chemotherapy drugs

Chemotherapy can cause negative physiological reactions in cancer patients and only partially suppress tumor growth (38). Cancer immunotherapy mediated by bacteria in combination with chemotherapy has the potential to overcome typical antitumor obstacles. Preclinical data have shown that specific lactic acid bacteria mixtures with adjuvant 5-fluorouracil chemotherapy inhibit 4T1 breast cancer tumor growth and associated side effects (186). More profoundly, interferon IFN-γ has been shown to promote attenuated Salmonella to kill mouse colon tumor cells by interrupting tumor recruitment concentration of granulocytes (187). Besides, IFN-γ stimulated M1-like macrophages and CD4(+) and CD8(+) T cells to infiltrate tumors to weaken bacterial immunogenicity (187). It has been discovered that the combination of chemotherapy and bacterial therapy is a promising antitumor immunotherapy.

Synergistic treatments for cancer have evolved thanks to bioengineering technology. For example, genetically modified bacterial protoplast-derived nanovesicles (PDNV) were used to load doxorubicin/idarubicin and deliver them to tumor tissue, precisely targeting chemotherapy for tumors (75). Another engineered Salmonella-driven drug delivery system allows natural ursolic acid UA to attract bacterial accumulation in CT26 colon tumors and accelerates tumor lysis (188). The natural antitumor immune activity of the bacteria was unaffected by these engineering techniques, which opens a new type of chemical-bacterial immunosynergistic therapy. This kind of preclinical combination therapy research fills the shortcomings of monotherapy and the safety has been fully confirmed, which is evolving into a clinical treatment for cancer patients.

### Promoted with immune checkpoint inhibitors

The current obvious defect of immune checkpoint inhibitors (ICIs) is that they bind to other immune cells expressing PD-L1, resulting in inefficient blockade of tumors and adverse effects. The shortcomings of the therapy could be reduced by the creation of particular engineered bacterial agents (208). For example, the self-blocking of PD-L1 in tumor cells was accomplished by a LyP1 polypeptide-modified outer membrane vesicle (LOMV) loaded with a PD-1 plasmid (158). Considerable preclinical research results to improve the treatment of bladder cancer are accumulating. For example, to promote dual immunity and the elimination of tumors, some researchers combined the use of attenuated mycobacterium MTBVAC with an anti-progressive cell death ligand (anti-PD-L1) (192). Furthermore, in therapeutically relevant cancer models, the inhibitory effects of targeted ultrasound-controlled bacteriotherapy *in situ* tumors have been established (126). What’s more attractive is that ultrasound-controlled E. coli can be combined and controlled the release of ICIs to optimize A20 tumor immunotherapy (201). The spatiotemporal controllability of ICIs in 4T1 tumors can also be improved by altering E. coli, which expresses photothermal melanin, and ICIs enhance melanin-induced immune response (200). But this combined technique may only be a useful tool in the treatment of solid malignancies. Furthermore, EcN was co-modified by ICIs, tumor-specific antigens, and polymeric dopamine to create agents for multimodal colon carcinoma immunotherapy. Then cytotoxic T lymphocytes were recognized to be activated by ICIs bound to bacterial surfaces (85). Dendritic cell maturation was directly induced by connected antigens, which in turn stimulate tumor-specific immune responses (85). Polydopamine exerted photothermal effects to stimulate the polarization of tumor-associated macrophages into pro-inflammatory phenotypic (85), hence the formation of a bacteria-based triple cancer immunotherapy platform. In summary, ICIs has demonstrated their value in synergistic therapy, mainly in the form of optimized and innovative bacterial antitumor immunotherapy.

### Combined with the oncolytic virus

Oncolytic viruses use the characteristics of unlimited reproduction of cancer cells to replicate offspring, thereby specifically targeting and destroying cancer cells. Oncolytic virotherapy and bacterial therapy are clinically translational components of novel cancer therapies (206), which both exert durable antitumor immune responses without little off-target toxicity (212). A new development in clinical treatment may result from the fusion of the two therapies. Traces back to the first preclinical example of using complementary combinations of microorganisms to improve tumor treatment outcomes, researchers used non-pathogenic E. coli expressing B18R to overcome innate immunity and deplete bioactive antiviral cytokines in the TEM, which greatly enhances the ability of subsequent intravenous hydro stomatitis virus (VSV) to infect and destroy tumors (203). In Murine Lewis Lung Carcinoma and HT29 adenocarcinoma model, evidence of effective treatment is a decrease in tumors and increased mouse survival. Besides, due to E. coil BL21’s ability to bind to OA through lipids, oncolytic adenovirus (OA) enrichment in non-small cell lung tumors is 170 times higher than that of intravenous naked OA (204). The ingenious combination of bacteria and viruses significantly enhances antitumor immunity. Similarly, the assembly of magnetostrophic bacteria and herpes virus (HSV1716) guided the virus to avoid the harm of neutralizing antibodies, promoting not only active viral targeting to tumors but also the enrichment of activated immune cells in tumors enabling dual immunological destruction of tumors (205). Overall, the unique role of microbiomes in cancer immunotherapy has the potential to optimize clinical roles.

## Safety and transformation evaluation for clinical applications

### Engineering sources and trends

**Figure 4:**
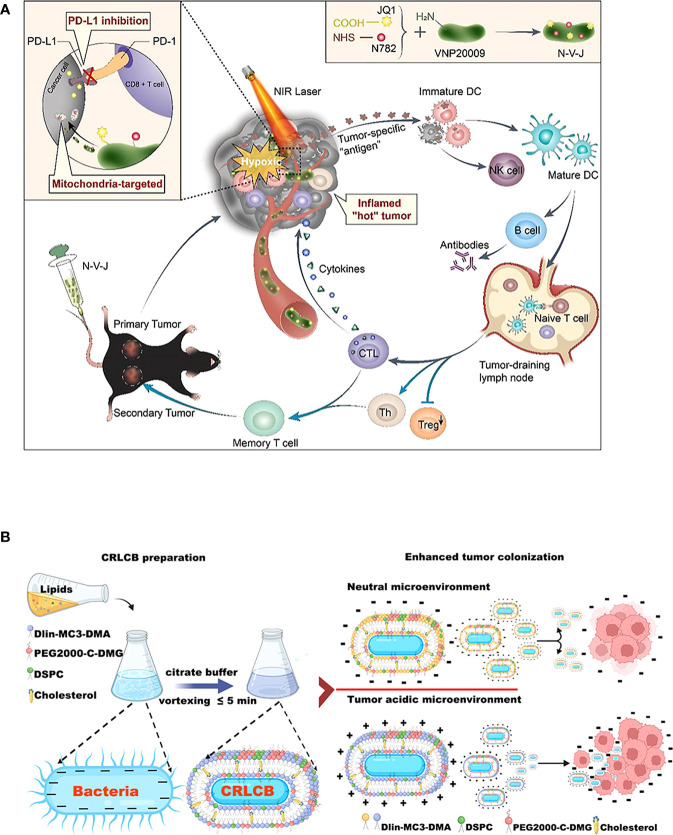
Schematic diagrams of engineered bacteria for antitumor immunotherapy. **(A)** Attenuated Salmonella VNP20009 as a hypoxic drug delivery system for loading the two small molecules, including a newly synthesized heptamethine cyanine dye NHS–N782 and a JQ-1 derivative, which assisted deep tumor photothermal therapy and enhanced immunotherapy. Reproduced with permission. (181) Copyright 2022, Elsevier Ltd. **(B)** Biointerfacial self-assembly DLin-MC3-DMA-containing lipids-coated E. coli transfer to positively charge in a tumor-acidic microenvironment, which benefits their internalization by tumor tissues. Reproduced with permission. (132) Copyright 2022, American Chemical Society.

The engineered bacterial model used for tumor immunotherapy is mainly derived from E. coli and Salmonella (213). These bacteria strains can tend to hypoxic TEM of their facultative anaerobic properties. When bacteria are enriched in the tumor region, attenuated treatment is usually used to reduce toxicity to the body (214), such as various attenuated S.typhimurium mutants (e.g., ΔppGpp, VNP20009, A1-R, SL7207, FAP-encoding S. typhimurium) formed by genetic engineering (168, 215–217). Based on the robust compatibility of the bacteria, they can then be modified to optimize tumor-specific localization (117), as an illustration, a metabolically engineered attenuated Salmonella treatment system was developed, which using the aggregation-induced emission (AIE) photosensitizer MA to localize Salmonella in tumor tissue (172). In addition, numerous natural immune adjuvants or immunotherapeutic agents have been identified for the tumor-suppressing immune environment, many of which are derived from the release of E. coli and Salmonella (195). For example, EcML, a mixture of 4’-monophosphoryl lipid A (MPLA) produced by engineered E. coli, activates DCs to initiate an antitumor adaptive immune response (196). The more striking optimization procedure is that of bacteria intended to be transformed into exogenous antitumor drug delivery vehicles (196) (**Figure 4A**). Bacterial immunotherapy is now more feasible in combination with other cancer treatments thanks to the engineered approach (191), for example, the addition of Salmonella immunotherapy has led to a safer prognosis for lymphoma patients treated with CHOP (190). Overall, S. typhimurium and E. coli dominate the antitumor development trend with unique advantages, including high tumor targeting specificity, deep tissue penetration, host immune system induction, and strong antitumor activity (132) (**Figure 4B**). Many bioengineering technologies further amplify the immunostimulation of bacteria to tumors, superimpose new antitumor immunity on bacteria, and may enhance the biosafety of bacterial immunotherapy agents during the clinical development process (218). The systems of engineered bacterial immunotherapy for cancer entering clinical trials are constantly being updated and are anticipated to produce effective results to optimize clinical treatment.

### Evaluation of colonization treatment modes

What cannot be ignored in clinical transformation is the effective persistence and toxicity control of therapeutic agents (219). In preclinical research, the modes of colonization of bacterial therapeutic agents are systemic colonization and local colonization. Systemic colonization, which includes oral and intravenous injections, enters systemic tissues and organs *via* gastrointestinal absorption or intravenous tracts (127, 220). Local colonization adopts intratumoral injection, subcutaneous injection, and other methods (143, 149, 197). Many bacterial agents, such as the exotoxin Pseudomonas rCCK8PE38 and the E. coli enterotoxin STa, are known to stimulate antitumor immune responses by releasing or expressing proteins (221), which have proven potential for cancer immunotherapy (152, 222). The layers of modification or coating result in an increase in the particle size of a bacterial therapeutic agent, for example, Mycobacterium tuberculosis loaded on Zn-, and Mg-containing tricalcium phosphates (TCPs) form potential immune-enhancing adjuvants with particle sizes up to 700 nm (138). Therefore, when systemic colonization occurs, it may exacerbate difficulties such as slowing the rate of tumor targeting and weakening the efficacy of killing them.

**Figure 5:**
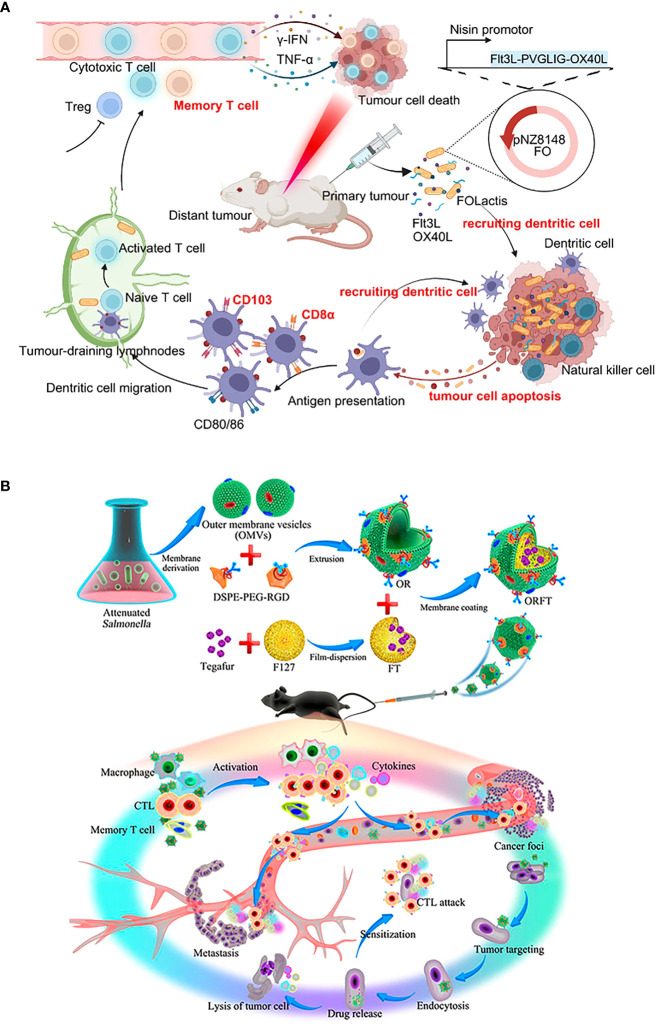
Schematic diagrams of the antitumor immune mechanism of locally colonized and systemic colonized bacterial therapeutics. **(A)** Intratumoral colonization of FOLactis enhances tumor immunotherapy through reprogramming the local tumor immune microenvironment and tumor-draining lymphnodes. Reproduced with permission (139). Copyright 2022, Nature Publishing Group. **(B)** Intravenous colonization of ORFT nanoparticles into melanoma-bearing mice could activate an antitumor response by the immunostimulatory of OMVs, further sensitize cancer cells to CTLs, and kill cancer cells directly through the effect of tegafur. Reproduced with permission (128). Copyright 2020, American Chemical Society.

It is equally critical to concentrate on biosafety assessment. Rather than local colonization, systemic colonization permits more bacterial treatments to accumulate in healthy tissues. For example, the lactococcal vaccine (FOLactis) peaked in the core of colon tumors after 3 hours of orthotopic colonization (139) (**Figure 5A**). But 3 hours after intravenous injection, engineered Salmonella vesicles-coated polymer nanodrugs accumulate mainly in tumors, livers, and kidneys (128) (**Figure 5B**). As a result, undesirable side effects such as an immunogenic attack, rejection, allergy, and toxicity are more prevalent. More consideration is the removal of the body after the therapeutic agent kills the tumor. Since local colonization is aimed at the tumor site, high-concentration doses are prone to produce local drug retention and thus evolve into toxic effects. Besides, it may be more susceptible to tolerability due to the rapid achievement of saturation therapeutic effects. To make the therapeutic agent uniformly and durably distributed in the tumor, a double-modified bacterium was designed to simultaneously control the localization of photothermal melanin and ICIs, to achieve a spatiotemporal and controllable dual immune antitumor effect (200). The minimization of adverse occurrences provides hints for solutions to practical issues in clinical translation.

### Applicability of tumor models

**Figure 6:**
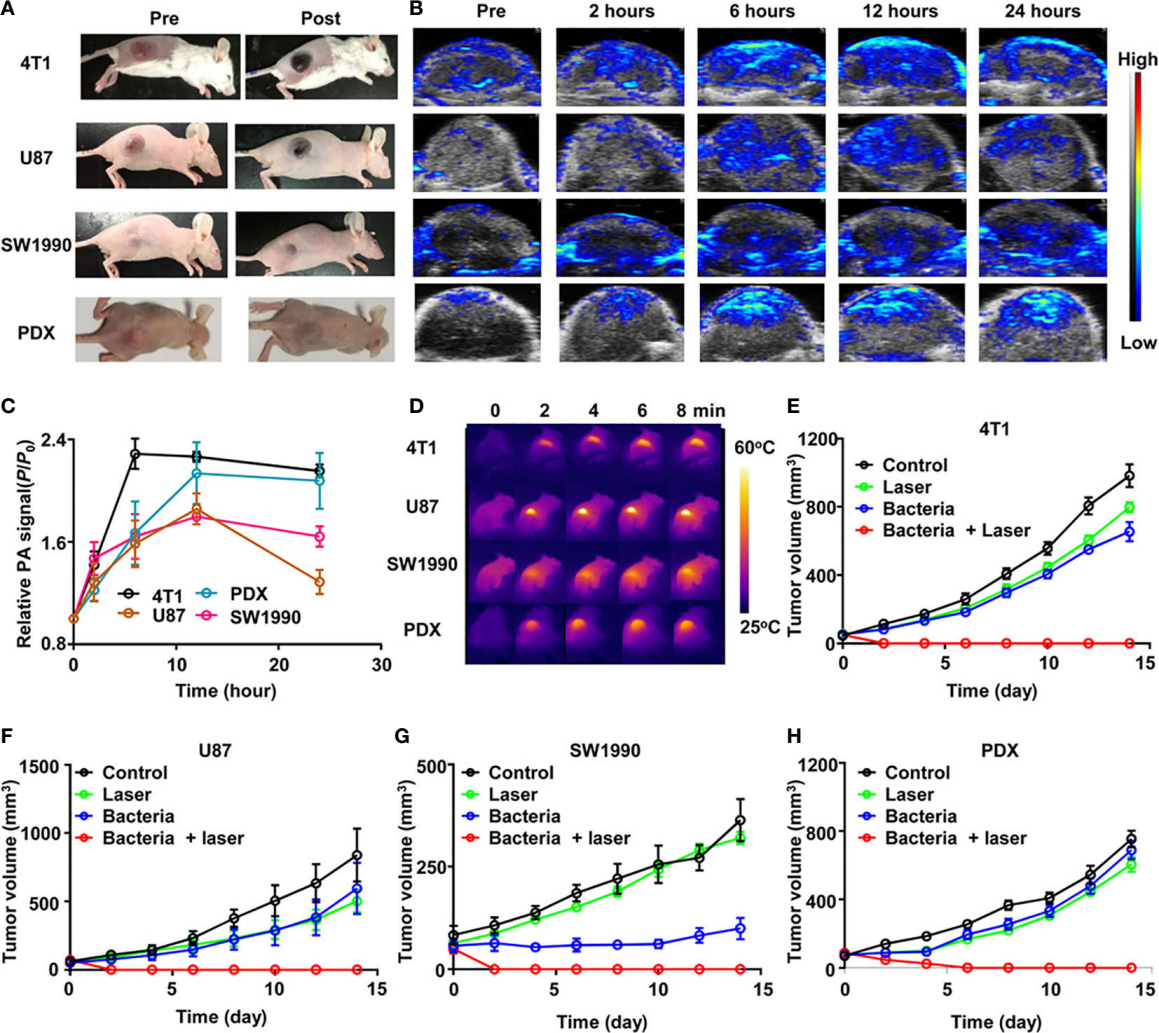
ΔppGpp S. typhimurium-triggered photothermal immunotherapy of multi-tumor models. **(A)** Photographs of BALB/c mice bearing 4T1, U87MG, SW1990, and patient-derived xenografts (PDX) tumors, before and after injection of bacteria at accordant does. **(B)** Representative PA images of different types of tumors on mice injected with bacteria at accordant do. **(C)** Quantification of relative PA signals of tumors on mice-injected bacteria. Data are presented as the mean ± SD. **(D)** Representative IR thermal images of the tumor-bearing mice injected with bacteria under 808-nm laser irradiation. **(E–H)** Tumor growth curves of mice bearing different kinds of tumor models, including 4T1 tumors **(E)**, U87MG tumors **(F)**, SW1990 tumors, **(G)**, and PDX tumors **(H)**, with different treatments indicated. Five mice were used for each group. Reproduced with permission (178). Copyright 2020. AMER ASSOC ADVANCEMENT SCIENCE.

Spontaneous tumor models do not form quickly or generate huge amounts of tumor material in a short period (150, 223). Besides, there are always discrepancies between artificial tumor models and actual malignancies, making extrapolating outcomes from animal clinical trials to people challenging. The use of orthotopic murine models humanized for CD40 and Fc-γ receptors demonstrated a promising new immune mechanism for the treatment of NMIBC, which has drawn significant attention to the humanization of preclinical animal models (164). To optimize the value of clinical translation, uniform model evaluation criteria should be established when using humanized and animal-derived tumor models to explore potential bacterial therapeutics (178) (**Figure 6**). In addition, multimodal animal models should be established to comprehensively evaluate tumor metastasis and recurrence, for example, while examining the curative effect in high metastatic orthotopic 4T1 tumor model and postsurgical recurrence model (224).

Whether in preclinical trials or clinical trials, cancers designed with novel bacterial therapeutics always favor solid tumors that are readily available and easy to achieve therapeutic effects (225). For example, mouse tumor models of 4T1, CT26, B16F10 (151), and gastrointestinal cancer are frequently used in preclinical studies (226), which have the advantages of relatively high induction rate, high degree of artificial control, and obvious efficacy. Recent ongoing clinical trials have selected patients with bladder cancer, prostate cancer, liver cancer, etc. (178, 227–229). Non-solid tumors are mainly hematopoietic system tumors that exist in the blood circulation, which are difficult to detect, grow quickly, and are ambiguously staged, so chemotherapy is currently the main treatment. This significantly restricts the application of common bacterial immunotherapy-based combination therapies for the treatment of hematological tumor models. Because of the extensive and complex immune mechanisms involved in various hematologic tumors such as leukemia and various lymphomas, the progression of a bacterial immune platform for nonsolid tumors remains critical.

### Production feasibility

One of the main challenges in achieving clinical production is to suppress the high immunogenicity of bacteria in humans. When combating tumors, it must be ensured that normal tissues, organs, and so forth are not affected. The Neiser-derived OMV of meningitidis is now regarded as safe and has the potential to be developed as the ideal antitumor vaccine vector (230). Besides, the absence of standardization of manufacturing settings and preparation processes makes bacteria prone to heterogeneity, limiting clinical application (231). The size, component, and content of the same batch of bacteria and derivatives varied greatly, for example, the component of OMV derived from Helicobacter pylori varied with the growth stage (232). Innovative quality control measures, such as better criteria for characterization parameters, are necessary for future bacteria-based medical products (231–233). Another significant issue is the control of antigen loading in bacterial vaccines. It is challenging to quantify the expression of endogenous antigens and the degree of modification of exogenous antigens (147). Achieving the optimal amount of antigen delivery is also challenging due to differences in vaccine colonization techniques and patient body composition (148). Therefore, the quantity of bacterial vaccine antigens should be accurately measured in preclinical evaluation, for example, the optimized auxotrophic Salmonella vector strain SF200 as a quantity of 5*106 injected into colorectal tumor models (136). Furthermore, promoting bacterial medical products needs to ensure economic viability. Poor extraction yield, a complicated manufacturing process, and the requirement for cutting-edge disinfection equipment are among the constraints restricting large-scale and inexpensive production of bacterial therapeutics (234). Fortunately, researchers have developed improvements such as detergent-free processes and continuous production methods (235–237). As the development of engineered bacterial therapeutics becomes more novel, it is essential to provide controllable stability and yield at the same time (238).

## Conclusion

Taken together, bacteria play an essential role in the tumor environment with their distinct intrinsic immune characteristics. They are crucial in triggering the antitumor response of non-specific cells and specific immune cells. Conventional natural bacterial immunotherapy has been improved by combining bioengineering techniques and multidisciplinary novel inventions. A series of preclinical antitumor bacterial vaccines and novel combination cancer therapies have shown versatile therapeutic effects (71). Several hurdles remain in the direct implementation of bacterial-based immunotherapies in clinical practice. To expedite the clinical translation of novel bacteria-based immunotherapy, we evaluated the engineering sources and trends of bacterial therapeutics, colonization treatment modes, suitable animal tumor models, as well as the feasibility of production. We expect that this review provides a reference for the optimization of bacterial-mediated tumor immunotherapy to enhance its value in clinical application.

## Author contributions

MZ, DX, and JW defined the focus of the review. MZ and JW summarized papers. MZ drafted the manuscript. All authors contributed to the article and approved the submitted version.

## References

[1] Bae J Park K Kim YM. Commensal microbiota and cancer immunotherapy: harnessing commensal bacteria for cancer therapy. Immune Netw (2022) 22(1):e3. doi: 10.4110/in.2022.22.e3 35291651 PMC8901697

[2] Slaney CY Kershaw MH. Challenges and opportunities for effective cancer immunotherapies. Cancers (Basel) (2020) 12(11):3164. doi: 10.3390/cancers12113164 33126513 PMC7693360

[3] Zhou CB Zhou YL Fang JY. Gut microbiota in cancer immune response and immunotherapy. Trends Cancer (2021) 7(7):647–60. doi: 10.1016/j.trecan.2021.01.010 33674230

[4] Huang X Pan J Xu F Shao B Wang Y Guo X. Bacteria-based cancer immunotherapy. Adv Sci (Weinh) (2021) 8(7):2003572. doi: 10.1002/advs.202003572 33854892 PMC8025040

[5] Tang Q Peng X Xu B Zhou X Chen J Cheng L. Current status and future directions of bacteria-based immunotherapy. Front Immunol (2022) 13:911783. doi: 10.3389/fimmu.2022.911783 35757741 PMC9226492

[6] Chen J Li T Liang J Huang Q Huang JD Ke Y. Current status of intratumour microbiome in cancer and engineered exogenous microbiota as a promising therapeutic strategy. BioMed Pharmacother (2022) 145:112443. doi: 10.1016/j.biopha.2021.112443 34847476

[7] Carlson RD Flickinger JC Jr. Snook AE. Talkin’ toxins: from coley’s to modern cancer immunotherapy. Toxins (Basel) (2020) 12(4):241. doi: 10.3390/toxins12040241 32283684 PMC7232517

[8] Kazmierczak-Siedlecka K Roviello G Catalano M Polom K. Gut microbiota modulation in the context of immune-related aspects of *Lactobacillus* spp. and *Bifidobacterium* spp. in gastrointestinal cancers. Nutrients (2021) 13(8):2674. doi: 10.3390/nu13082674 34444834 PMC8401094

[9] McCulloch JA Davar D Rodrigues RR Badger JH Fang JR Cole AM. Intestinal microbiota signatures of clinical response and immune-related adverse events in melanoma patients treated with anti-PD-1. Nat Med (2022) 28(3):545–56. doi: 10.1038/s41591-022-01698-2 PMC10246505 35228752

[10] Osuna-Perez J Garcia-Ferreras R Veiga E. From cellular microbiology to bacteria-based next generations of cancer immunotherapies. Cell Microbiol (2020) 22(4):e13187. doi: 10.1111/cmi.13187 32185897

[11] Khosravi M Khazaeil K KhademiMoghadam F. Triggering of the immune response to MCF7 cell line using conjugated antibody with bacterial antigens: in-vitro and in-vivo study. PloS One (2022) 17(10):e0275776. doi: 10.1371/journal.pone.0275776 36206297 PMC9543947

[12] Li M Zhou H Yang C Wu Y Zhou X Liu H. Bacterial outer membrane vesicles as a platform for biomedical applications: an update. J Control Release (2020) 323:253–68. doi: 10.1016/j.jconrel.2020.04.031 32333919

[13] Cao Z Liu J. Bacteria and bacterial derivatives as drug carriers for cancer therapy. J Control Release (2020) 326:396–407. doi: 10.1016/j.jconrel.2020.07.009 32681947

[14] Chen Y Wu FH Wu PQ Xing HY Ma T. The role of the tumor microbiome in tumor development and its treatment. Front Immunol (2022) 13:935846. doi: 10.3389/fimmu.2022.935846 35911695 PMC9334697

[15] Lu H Wang Q Liu W Wen Z Li Y. Precision strategies for cancer treatment by modifying the tumor-related bacteria. Appl Microbiol Biotechnol (2021) 105(16-17):6183–97. doi: 10.1007/s00253-021-11491-9 34402938

[16] Oladejo M Paterson Y Wood LM. Clinical experience and recent advances in the development of listeria-based tumor immunotherapies. Front Immunol (2021) 12:642316. doi: 10.3389/fimmu.2021.642316 33936058 PMC8081050

[17] Yang Z Zhu Y Dong Z Hao Y Wang C Li Q. Engineering bioluminescent bacteria to boost photodynamic therapy and systemic anti-tumor immunity for synergistic cancer treatment. Biomaterials (2022) 281:121332. doi: 10.1016/j.biomaterials.2021.121332 35066286

[18] Sanmamed MF Chen L. A paradigm shift in cancer immunotherapy: from enhancement to normalization. Cell (2018) 175(2):313–26. doi: 10.1016/j.cell.2018.09.035 PMC6538253 30290139

[19] Chavez-Arroyo A Portnoy DA. Why is listeria monocytogenes such a potent inducer of CD8+ T-cells? Cell Microbiol (2020) 22(4):e13175. doi: 10.1111/cmi.13175 32185899 PMC7100999

[20] Sepich-Poore GD Zitvogel L Straussman R Hasty J Wargo JA Knight R. The microbiome and human cancer. Science (2021) 371(6536):eabc4552. doi: 10.1126/science.abc4552 33766858 PMC8767999

[21] Duong MT Qin Y You and J.J. Min SH. Bacteria-cancer interactions: bacteria-based cancer therapy. Exp Mol Med (2019) 51(12):1–15. doi: 10.1038/s12276-019-0297-0 PMC6906302 31827064

[22] Badie F Ghandali M Tabatabaei SA Safari M Khorshidi A Shayestehpour M. Use of salmonella bacteria in cancer therapy: direct, drug delivery and combination approaches. Front Oncol (2021) 11:624759. doi: 10.3389/fonc.2021.624759 33738260 PMC7960920

[23] Yaghoubi A Khazaei M Jalili S Hasanian SM Avan A Soleimanpour S. Bacteria as a double-action sword in cancer. Biochim Biophys Acta Rev Cancer (2020) 1874(1):188388. doi: 10.1016/j.bbcan.2020.188388 32589907

[24] Li Y Zhao R Cheng K Zhang K Wang Y Zhang Y. Bacterial outer membrane vesicles presenting programmed death 1 for improved cancer immunotherapy via immune activation and checkpoint inhibition. ACS Nano (2020), 16698–711. doi: 10.1021/acsnano.0c03776 33232124

[25] Hoffman RM Zhao M. Methods for the development of tumor-targeting bacteria. Expert Opin Drug Discov (2014) 9(7):741–50. doi: 10.1517/17460441.2014.916270 24949888

[26] Al-Saafeen BH Fernandez-Cabezudo MJ Al-Ramadi BK. Integration of salmonella into combination cancer therapy. Cancers (Basel) (2021) 13(13):3228. doi: 10.3390/cancers13133228 34203478 PMC8269432

[27] Guo Y Chen Y Liu X Min JJ Tan W Zheng JH. Targeted cancer immunotherapy with genetically engineered oncolytic salmonella typhimurium. Cancer Lett (2020) 469:102–10. doi: 10.1016/j.canlet.2019.10.033 31666180

[28] Chen MC Pangilinan CR Lee CH. Salmonella breaks tumor immune tolerance by downregulating tumor programmed death-ligand 1 expression. Cancers (Basel) (2019) 12(1):57. doi: 10.3390/cancers12010057 31878272 PMC7017279

[29] Al-Saafeen BH Al-Sbiei A Bashir G Mohamed YA Masad RJ Fernandez-Cabezudo MJ. Attenuated salmonella potentiate PD-L1 blockade immunotherapy in a preclinical model of colorectal cancer. Front Immunol (2022) 13:1017780. doi: 10.3389/fimmu.2022.1017780 36605208 PMC9807881

[30] Wang D Wei X Kalvakolanu DV Guo B Zhang L. Perspectives on oncolytic salmonella in cancer immunotherapy-a promising strategy. Front Immunol (2021) 12:615930. doi: 10.3389/fimmu.2021.615930 33717106 PMC7949470

[31] Xie YJ Huang M Li D Hou JC Liang HH Nasim AA. Bacteria-based nanodrug for anticancer therapy. Pharmacol Res (2022) 182:106282. doi: 10.1016/j.phrs.2022.106282 35662630

[32] Merlano MC Granetto C Fea E Ricci V Garrone O. Heterogeneity of colon cancer: from bench to bedside. ESMO Open (2017) 2(3):e000218. doi: 10.1136/esmoopen-2017-000218 29209524 PMC5703395

[33] Owens B. Gut bacteria link to immunotherapy sparks interest. Nat Biotechnol (2018) 36(2):121–3. doi: 10.1038/nbt0218-121 29406499

[34] Fessler J Matson V Gajewski TF. Exploring the emerging role of the microbiome in cancer immunotherapy. J Immunother Cancer (2019) 7(1):108. doi: 10.1186/s40425-019-0574-4 30995949 PMC6471869

[35] DeStefano Shields CE White JR Chung L Wenzel A Hicks JL Tam AJ. Bacterial-driven inflammation and mutant BRAF expression combine to promote murine colon tumorigenesis that is sensitive to immune checkpoint therapy. Cancer Discov (2021) 11(7):1792–807. doi: 10.1158/2159-8290.CD-20-0770 PMC8295175 33632774

[36] Pitt JM Vetizou M Waldschmitt N Kroemer G Chamaillard M Boneca IG. Fine-tuning cancer immunotherapy: optimizing the gut microbiome. Cancer Res (2016) 76(16):4602–7. doi: 10.1158/0008-5472.CAN-16-0448 27474734

[37] Dey N Ciorba MA. Probiotic gut bacteria enhance cancer immunotherapy in a mouse model of melanoma. Gastroenterology (2016) 151(1):206–7. doi: 10.1053/j.gastro.2016.05.015 27238844

[38] Shi L Sheng J Wang M Luo H Zhu J Zhang B. Combination therapy of TGF-beta blockade and commensal-derived probiotics provides enhanced antitumor immune response and tumor suppression. Theranostics (2019) 9(14):4115–29. doi: 10.7150/thno.35131 PMC6592171 31281535

[39] Badgeley A Anwar H Modi K Murphy P Lakshmikuttyamma A. Effect of probiotics and gut microbiota on anti-cancer drugs: mechanistic perspectives. Biochim Biophys Acta Rev Cancer (2021) 1875(1):188494. doi: 10.1016/j.bbcan.2020.188494 33346129

[40] Nasr R Shamseddine A Mukherji D Nassar F Temraz S. The crosstalk between microbiome and immune response in gastric cancer. Int J Mol Sci (2020) 21(18):6568. doi: 10.3390/ijms21186586 32916853 PMC7556019

[41] Gao G Ma T Zhang T Jin H Li Y Kwok LY. Adjunctive probiotic lactobacillus rhamnosus probio-M9 administration enhances the effect of anti-PD-1 antitumor therapy via restoring antibiotic-disrupted gut microbiota. Front Immunol (2021) 12:772532. doi: 10.3389/fimmu.2021.772532 34970262 PMC8712698

[42] Montalban-Arques A Katkeviciute E Busenhart P Bircher A Wirbel J Zeller G. Commensal clostridiales strains mediate effective anti-cancer immune response against solid tumors. Cell Host Microbe (2021) 29(10):1573–1588 e1577. doi: 10.1016/j.chom.2021.08.001 34453895

[43] Ansaldo E Slayden LC Ching KL Koch MA Wolf NK Plichta DR. Akkermansia muciniphila induces intestinal adaptive immune responses during homeostasis. Science (2019) 364(6446):1179–84. doi: 10.1126/science.aaw7479 PMC6645389 31221858

[44] Anker JF Naseem AF Mok H Schaeffer AJ Abdulkadir SA Thumbikat P. Multi-faceted immunomodulatory and tissue-tropic clinical bacterial isolate potentiates prostate cancer immunotherapy. Nat Commun (2018) 9(1):1591. doi: 10.1038/s41467-018-03900-x 29686284 PMC5913311

[45] King C. Tfh cells set the stage for tumor control. Immunity (2021) 54(12):2690–2. doi: 10.1016/j.immuni.2021.11.013 34910936

[46] Tomita Y Goto Y Sakata S Imamura K Minemura A Oka K. Clostridium butyricum therapy restores the decreased efficacy of immune checkpoint blockade in lung cancer patients receiving proton pump inhibitors. Oncoimmunology (2022) 11(1):2081010. doi: 10.1080/2162402X.2022.2081010 35655708 PMC9154751

[47] Yang X Guo Y Chen C Shao B Zhao L Zhou Q. Interaction between intestinal microbiota and tumour immunity in the tumour microenvironment. Immunology (2021) 164(3):476–93. doi: 10.1111/imm.13397 PMC8517597 34322877

[48] Granicka LH Borkowska M Grzeczkowicz A Stachowiak R Szklarczyk M Bielecki J. The targeting nanothin polyelectrolyte shells in system with immobilized bacterial cells for antitumor factor production. J BioMed Mater Res A (2014) 102(8):2662–8. doi: 10.1002/jbm.a.34936 23982999

[49] Zheng JH Nguyen VH Jiang SN Park SH Tan W Hong SH. Two-step enhanced cancer immunotherapy with engineered salmonella typhimurium secreting heterologous flagellin. Sci Transl Med (2017) 9(376):eaak9537. doi: 10.1126/scitranslmed.aak9537 28179508

[50] Crunkhorn S. Cancer: bacterium-based immunotherapy. Nat Rev Drug Discov (2017) 16(4):240. doi: 10.1038/nrd.2017.54 28356590

[51] Hajam IA Dar PA Shahnawaz I Jaume JC Lee JH. Bacterial flagellin-a potent immunomodulatory agent. Exp Mol Med (2017) 49(9):e373. doi: 10.1038/emm.2017.172 28860663 PMC5628280

[52] Binder DC Wainwright DA. The boosting potential of bacteria in cancer immunotherapy. Trends Mol Med (2017) 23(7):580–2. doi: 10.1016/j.molmed.2017.05.008 PMC5515260 28583420

[53] Shiga M Miyazaki J Tanuma K Nagumo Y Yoshino T Kandori S. The liposome of trehalose dimycolate extracted from m. bovis BCG induces antitumor immunity via the activation of dendritic cells and CD8(+) T cells. Cancer Immunol Immunother (2021) 70(9):2529–43. doi: 10.1007/s00262-021-02870-2 PMC10992466 33570675

[54] Gregg KA Harberts E Gardner FM Pelletier MR Cayatte C Yu L. Rationally designed TLR4 ligands for vaccine adjuvant discovery. mBio (2017) 8(3):e00492–17. doi: 10.1128/mBio.00492-17 PMC5424205 28487429

[55] Zhang M Kim JA Huang AY. Optimizing tumor microenvironment for cancer immunotherapy: beta-Glucan-Based nanoparticles. Front Immunol (2018) 9:341. doi: 10.3389/fimmu.2018.00341 29535722 PMC5834761

[56] Guryanova SV Khaitov RM. Strategies for using muramyl peptides - modulators of innate immunity of bacterial origin - in medicine. Front Immunol (2021) 12:607178. doi: 10.3389/fimmu.2021.607178 33959120 PMC8093441

[57] Viaud S Daillere R Boneca IG Lepage P Pittet MJ Ghiringhelli F. Harnessing the intestinal microbiome for optimal therapeutic immunomodulation. Cancer Res (2014) 74(16):4217–21. doi: 10.1158/0008-5472.CAN-14-0987 PMC6700746 25074615

[58] Song W Tiruthani K Wang Y Shen L Hu M Dorosheva O. Trapping of lipopolysaccharide to promote immunotherapy against colorectal cancer and attenuate liver metastasis. Adv Mater (2018) 30(52):e1805007. doi: 10.1002/adma.201805007 30387230 PMC6580426

[59] Felgner S Kocijancic D Frahm M Curtiss R Erhardt M Weiss S. Optimizing salmonella enterica serovar typhimurium for bacteria-mediated tumor therapy. Gut Microbes (2016) 7(2):171–7. doi: 10.1080/19490976.2016.1155021 PMC4856459 26939530

[60] Lim J Koh VHQ Cho SSL Periaswamy B Choi DPS Vacca M. Harnessing the immunomodulatory properties of bacterial ghosts to boost the anti-mycobacterial protective immunity. Front Immunol (2019) 10:2737. doi: 10.3389/fimmu.2019.02737 31824511 PMC6883722

[61] Groza D Gehrig S Kudela P Holcmann M Pirker C Dinhof C. Bacterial ghosts as adjuvant to oxaliplatin chemotherapy in colorectal carcinomatosis. Oncoimmunology (2018) 7(5):e1424676. doi: 10.1080/2162402X.2018.1424676 29721389 PMC5927527

[62] Luteijn RD Zaver SA Gowen BG Wyman SK Garelis NE Onia L. SLC19A1 transports immunoreactive cyclic dinucleotides. Nature (2019) 573(7774):434–8. doi: 10.1038/s41586-019-1553-0 PMC6785039 31511694

[63] Sekurova ON Schneider O Zotchev SB. Novel bioactive natural products from bacteria via bioprospecting, genome mining and metabolic engineering. Microb Biotechnol (2019) 12(5):828–44. doi: 10.1111/1751-7915.13398 PMC6680616 30834674

[64] Johnson CH Spilker ME Goetz L Peterson SN Siuzdak G. Metabolite and microbiome interplay in cancer immunotherapy. Cancer Res (2016) 76(21):6146–52. doi: 10.1158/0008-5472.CAN-16-0309 PMC5093024 27729325

[65] Grenda A Krawczyk P. Cancer trigger or remedy: two faces of the human microbiome. Appl Microbiol Biotechnol (2021) 105(4):1395–405. doi: 10.1007/s00253-021-11125-0 33492450

[66] Colombani T Haudebourg T Decossas M Lambert O Ada Da Silva G Altare F. Lipidic aminoglycoside derivatives: a new class of immunomodulators inducing a potent innate immune stimulation. Adv Sci (Weinh) (2019) 6(16):1900288. doi: 10.1002/advs.201900288 31453059 PMC6702646

[67] Mazor R King EM Pastan I. Strategies to reduce the immunogenicity of recombinant immunotoxins. Am J Pathol (2018) 188(8):1736–43. doi: 10.1016/j.ajpath.2018.04.016 PMC6099333 29870741

[68] Mager LF Burkhard R Pett N Cooke NCA Brown K Ramay H. Microbiome-derived inosine modulates response to checkpoint inhibitor immunotherapy. Science (2020) 369(6510):1481–9. doi: 10.1126/science.abc3421 32792462

[69] Yang C Cui M Zhang Y Pan H Liu J Wang S. Upconversion optogenetic micro-nanosystem optically controls the secretion of light-responsive bacteria for systemic immunity regulation. Commun Biol (2020) 3(1):561. doi: 10.1038/s42003-020-01287-4 33037315 PMC7547716

[70] Gao J Su Y Wang Z. Engineering bacterial membrane nanovesicles for improved therapies in infectious diseases and cancer. Adv Drug Deliv Rev (2022) 186:114340. doi: 10.1016/j.addr.2022.114340 35569561 PMC9899072

[71] Zhai Y Ma Y Pang B Zhang J Li Y Rui Y. A cascade targeting strategy based on modified bacterial vesicles for enhancing cancer immunotherapy. J Nanobiotechnol (2021) 19(1):434. doi: 10.1186/s12951-021-01193-9 PMC8686283 34930285

[72] Fazal S Lee R. Biomimetic bacterial membrane vesicles for drug delivery applications. Pharmaceutics (2021) 13(9):1430. doi: 10.3390/pharmaceutics13091430 34575506 PMC8468068

[73] Long Q Zheng P Zheng X Li W Hua L Yang Z. Engineered bacterial membrane vesicles are promising carriers for vaccine design and tumor immunotherapy. Adv Drug Deliv Rev (2022) 186:114321. doi: 10.1016/j.addr.2022.114321 35533789

[74] Kuerban K Gao X Zhang H Liu J Dong M Wu L. Doxorubicin-loaded bacterial outer-membrane vesicles exert enhanced anti-tumor efficacy in non-small-cell lung cancer. Acta Pharm Sin B (2020) 10(8):1534–48. doi: 10.1016/j.apsb.2020.02.002 PMC7488491 32963948

[75] Kim OY Dinh NT Park HT Choi SJ Hong K Gho YS. Bacterial protoplast-derived nanovesicles for tumor targeted delivery of chemotherapeutics. Biomaterials (2017) 113:68–79. doi: 10.1016/j.biomaterials.2016.10.037 27810643

[76] Aytar Celik P Derkus B Erdogan K Barut D Blaise Manga E Yildirim Y. Bacterial membrane vesicle functions, laboratory methods, and applications. Biotechnol Adv (2022) 54:107869. doi: 10.1016/j.biotechadv.2021.107869 34793882

[77] Zhao X Zhao R Nie G. Nanocarriers based on bacterial membrane materials for cancer vaccine delivery. Nat Protoc (2022) 17(10):2240–74. doi: 10.1038/s41596-022-00713-7 35879454

[78] Wang S Gao J Li M Wang L Wang Z. A facile approach for development of a vaccine made of bacterial double-layered membrane vesicles (DMVs). Biomaterials (2018) 187:28–38. doi: 10.1016/j.biomaterials.2018.09.042 30292939 PMC6205512

[79] Kim OY Park HT Dinh NTH Choi SJ Lee J Kim JH. Bacterial outer membrane vesicles suppress tumor by interferon-gamma-mediated antitumor response. Nat Commun (2017) 8(1):626. doi: 10.1038/s41467-017-00729-8 28931823 PMC5606984

[80] Park KS Svennerholm K Crescitelli R Lasser C Gribonika I Lotvall J. Synthetic bacterial vesicles combined with tumour extracellular vesicles as cancer immunotherapy. J Extracell Vesicles (2021) 10(9):e12120. doi: 10.1002/jev2.12120 34262675 PMC8254025

[81] Byrne WL Murphy CT Cronin M Wirth T Tangney M. Bacterial-mediated DNA delivery to tumour associated phagocytic cells. J Control Release (2014) 196:384–93. doi: 10.1016/j.jconrel.2014.10.030 25466954

[82] Lee CH Lin YH Hsieh JL Chen MC Kuo WL. A polymer coating applied to salmonella prevents the binding of salmonella-specific antibodies. Int J Cancer (2013) 132(3):717–25. doi: 10.1002/ijc.27700 22736433

[83] Tan QL Zhou CY Cheng L Luo M Liu CP Xu WX. Immunotherapy of bacillus Calmette−Guerin by targeting macrophages against bladder cancer in a NOD/scid IL2Rg−/− mouse model. Mol Med Rep (2020) 22(1):362–70. doi: 10.3892/mmr.2020.11090 PMC7248479 32319653

[84] Wei B Pan J Yuan R Shao B Wang Y Guo X. Polarization of tumor-associated macrophages by nanoparticle-loaded escherichia coli combined with immunogenic cell death for cancer immunotherapy. Nano Lett (2021) 21(10):4231–40. doi: 10.1021/acs.nanolett.1c00209 33998789

[85] Li J Xia Q Guo H Fu Z Liu Y Lin S. Decorating bacteria with triple immune nanoactivators generates tumor-resident living immunotherapeutics. Angew Chem Int Ed Engl (2022) 61(27):e202202409. doi: 10.1002/anie.202202409 35403784

[86] Luo ZW Xia K Liu YW Liu JH Rao SS Hu XK. Extracellular vesicles from akkermansia muciniphila elicit antitumor immunity against prostate cancer via modulation of CD8(+) T cells and macrophages. Int J Nanomed (2021) 16:2949–63. doi: 10.2147/IJN.S304515 PMC8068512 33907401

[87] Kuhn S Hyde EJ Yang J Rich FJ Harper JL Kirman JR. Increased numbers of monocyte-derived dendritic cells during successful tumor immunotherapy with immune-activating agents. J Immunol (2013) 191(4):1984–92. doi: 10.4049/jimmunol.1301135 23858033

[88] Jeong H Lee SY Seo H Kim DH Lee D Kim BJ. Potential of mycobacterium tuberculosis chorismate mutase (Rv1885c) as a novel TLR4-mediated adjuvant for dendritic cell-based cancer immunotherapy. Oncoimmunology (2022) 11(1):2023340. doi: 10.1080/2162402X.2021.2023340 35083095 PMC8786331

[89] Chiang CY Wu CC Chen YJ Liu SJ Leng CH Chen HW. Delivery of antigen to CD8(+) dendritic cells by fusing antigen with formyl peptide receptor-like 1 inhibitor protein induces antitumor immunity. Front Immunol (2019) 10:1839. doi: 10.3389/fimmu.2019.01839 31428106 PMC6688046

[90] Shi Y Zheng W Yang K Harris KG Ni K Xue L. Intratumoral accumulation of gut microbiota facilitates CD47-based immunotherapy via STING signaling. J Exp Med (2020) 217(5):e20192282. doi: 10.1084/jem.20192282 32142585 PMC7201921

[91] Michalek J Hezova R Turanek-Knotigova P Gabkova J Strioga M Lubitz W. Oncolysate-loaded escherichia coli bacterial ghosts enhance the stimulatory capacity of human dendritic cells. Cancer Immunol Immunother (2017) 66(2):149–59. doi: 10.1007/s00262-016-1932-4 PMC11029152 27864613

[92] Coria LM Ibanez AE Tkach M Sabbione F Bruno L Carabajal MV. A brucella spp. protease inhibitor limits antigen lysosomal proteolysis, increases cross-presentation, and enhances CD8+ T cell responses. J Immunol (2016) 196(10):4014–29. doi: 10.4049/jimmunol.1501188 27084100

[93] Portevin D Young D. Natural killer cell cytokine response to m. bovis BCG is associated with inhibited proliferation, increased apoptosis and ultimate depletion of NKp44(+)CD56(bright) cells. PloS One (2013) 8(7):e68864. doi: 10.1371/journal.pone.0068864 23874793 PMC3715299

[94] Esteso G Aguilo N Julian E Ashiru O Ho MM Martin C. Natural killer anti-tumor activity can be achieved by in vitro incubation with heat-killed BCG. Front Immunol (2021) 12:622995. doi: 10.3389/fimmu.2021.622995 33708215 PMC7940681

[95] Lin Q Rong L Jia X Li R Yu B Hu J. IFN-gamma-dependent NK cell activation is essential to metastasis suppression by engineered salmonella. Nat Commun (2021) 12(1):2537. doi: 10.1038/s41467-021-22755-3 33953170 PMC8099885

[96] Wu J He B Miao M Han X Dai H Dou H. Enhancing natural killer cell-mediated cancer immunotherapy by the biological macromolecule nocardia rubra cell-wall skeleton. Pathol Oncol Res (2022) 28:1610555. doi: 10.3389/pore.2022.1610555 36110249 PMC9468226

[97] DeClue AE Axiak-Bechtel SM Zhang Y Saha S Zhang L Tung D. Immune response to c. novyi-NT immunotherapy. Vet Res (2018) 49(1):38. doi: 10.1186/s13567-018-0531-0 29690928 PMC5937821

[98] Xia Y Chen B Shao X Xiao W Qian L Ding Y. Treatment with a fusion protein of the extracellular domains of NKG2D to IL-15 retards colon cancer growth in mice. J Immunother (2014) 37(5):257–66. doi: 10.1097/CJI.0000000000000033 24810637

[99] Zitvogel L Kroemer G. Cross-reactivity between microbial and tumor antigens. Curr Opin Immunol (2022) 75:102171. doi: 10.1016/j.coi.2022.102171 35219942

[100] Xue LJ Mao XB Liu XB Gao H Chen YN Dai TT. Activation of CD3(+) T cells by helicobacter pylori DNA vaccines in potential immunotherapy of gastric carcinoma. Cancer Biol Ther (2019) 20(6):866–76. doi: 10.1080/15384047.2019.1579957 PMC6605983 30786815

[101] Chen Z Ozbun L Chong N Wallecha A Berzofsky JA Khleif SN. Episomal expression of truncated listeriolysin O in LmddA-LLO-E7 vaccine enhances antitumor efficacy by preferentially inducing expansions of CD4+FoxP3- and CD8+ T cells. Cancer Immunol Res (2014) 2(9):911–22. doi: 10.1158/2326-6066.CIR-13-0197 PMC4160031 24872025

[102] Stern C Kasnitz N Kocijancic D Trittel S Riese P Guzman CA. Induction of CD4(+) and CD8(+) anti-tumor effector T cell responses by bacteria mediated tumor therapy. Int J Cancer (2015) 137(8):2019–28. doi: 10.1002/ijc.29567 25868911

[103] Deng W Lira V Hudson TE Lemmens EE Hanson WG Flores R. Recombinant listeria promotes tumor rejection by CD8(+) T cell-dependent remodeling of the tumor microenvironment. Proc Natl Acad Sci USA (2018) 115(32):8179–84. doi: 10.1073/pnas.1801910115 PMC6094133 30038013

[104] Antonelli AC Binyamin A Hohl TM Glickman MS Redelman-Sidi G. Bacterial immunotherapy for cancer induces CD4-dependent tumor-specific immunity through tumor-intrinsic interferon-gamma signaling. Proc Natl Acad Sci USA (2020) 117(31):18627–37. doi: 10.1073/pnas.2004421117 PMC7414065 32680964

[105] Vetizou M Pitt JM Daillere R Lepage P Waldschmitt N Flament C. Anticancer immunotherapy by CTLA-4 blockade relies on the gut microbiota. Science (2015) 350(6264):1079–84. doi: 10.1126/science.aad1329 PMC4721659 26541610

[106] Bessell CA Isser A Havel JJ Lee S Bell DR Hickey JW. Commensal bacteria stimulate antitumor responses via T cell cross-reactivity. JCI Insight (2020) 5(8):e135597. doi: 10.1172/jci.insight.135597 32324171 PMC7205429

[107] Zhu G Su H Johnson CH Khan SA Kluger H Lu L. Intratumour microbiome associated with the infiltration of cytotoxic CD8+ T cells and patient survival in cutaneous melanoma. Eur J Cancer (2021) 151:25–34. doi: 10.1016/j.ejca.2021.03.053 33962358 PMC8184628

[108] Canale FP Basso C Antonini G Perotti M Li N Sokolovska A. Metabolic modulation of tumours with engineered bacteria for immunotherapy. Nature (2021) 598(7882):662–6. doi: 10.1038/s41586-021-04003-2 34616044

[109] Chowdhury S Castro S Coker C Hinchliffe TE Arpaia N Danino T. Programmable bacteria induce durable tumor regression and systemic antitumor immunity. Nat Med (2019) 25(7):1057–63. doi: 10.1038/s41591-019-0498-z PMC6688650 31270504

[110] Murakami T Hiroshima Y Zhang Y Zhao M Kiyuna T Hwang HK. Tumor-targeting salmonella typhimurium A1-r promotes tumoricidal CD8(+) T cell tumor infiltration and arrests growth and metastasis in a syngeneic pancreatic-cancer orthotopic mouse model. J Cell Biochem (2018) 119(1):634–9. doi: 10.1002/jcb.26224 28628234

[111] Moreno VM Baeza A. Bacteria as nanoparticle carriers for immunotherapy in oncology. Pharmaceutics (2022) 14(4):784. doi: 10.3390/pharmaceutics14040784 35456618 PMC9027800

[112] Mowday AM Guise CP Ackerley DF Minton NP Lambin P Dubois LJ. Advancing clostridia to clinical trial: past lessons and recent progress. Cancers (Basel) (2016) 8(7):63. doi: 10.3390/cancers8070063 27367731 PMC4963805

[113] Khallouf H Grabowska AK Riemer AB. Therapeutic vaccine strategies against human papillomavirus. Vaccines (Basel) (2014) 2(2):422–62. doi: 10.3390/vaccines2020422 PMC4494257 26344626

[114] Schmitz-Winnenthal FH Hohmann N Niethammer AG Friedrich T Lubenau H Springer M. Anti-angiogenic activity of VXM01, an oral T-cell vaccine against VEGF receptor 2, in patients with advanced pancreatic cancer: a randomized, placebo-controlled, phase 1 trial. Oncoimmunology (2015) 4(4):e1001217. doi: 10.1080/2162402X.2014.1001217 26137397 PMC4485742

[115] Vendrell A Mongini C Gravisaco MJ Canellada A Tesone AI Goin JC. An oral salmonella-based vaccine inhibits liver metastases by promoting tumor-specific T-Cell-Mediated immunity in celiac and portal lymph nodes: a preclinical study. Front Immunol (2016) 7:72. doi: 10.3389/fimmu.2016.00072 26973649 PMC4771756

[116] Hu Q Wu M Fang C Cheng C Zhao M Fang W. Engineering nanoparticle-coated bacteria as oral DNA vaccines for cancer immunotherapy. Nano Lett (2015) 15(4):2732–9. doi: 10.1021/acs.nanolett.5b00570 25806599

[117] Stermann A Huebener N Seidel D Fest S Eschenburg G Stauder M. Targeting of MYCN by means of DNA vaccination is effective against neuroblastoma in mice. Cancer Immunol Immunother (2015) 64(10):1215–27. doi: 10.1007/s00262-015-1733-1 PMC11028418 26076666

[118] Kitagawa K Gonoi R Tatsumi M Kadowaki M Katayama T Hashii Y. Preclinical development of a WT1 oral cancer vaccine using a bacterial vector to treat castration-resistant prostate cancer. Mol Cancer Ther (2019) 18(5):980–90. doi: 10.1158/1535-7163.MCT-18-1105 30824610

[119] Kitagawa K Oda T Saito H Araki A Gonoi R Shigemura K. Development of oral cancer vaccine using recombinant bifidobacterium displaying wilms’ tumor 1 protein. Cancer Immunol Immunother (2017) 66(6):787–98. doi: 10.1007/s00262-017-1984-0 PMC11028424 28299466

[120] Shirakawa T Kitagawa K. Antitumor effect of oral cancer vaccine with bifidobacterium delivering WT1 protein to gut immune system is superior to WT1 peptide vaccine. Hum Vaccin Immunother (2018) 14(1):159–62. doi: 10.1080/21645515.2017.1382787 PMC5791589 29048978

[121] Taghinezhad SS Mohseni AH Keyvani H Razavilar V. Protection against human papillomavirus type 16-induced tumors in C57BL/6 mice by mucosal vaccination with lactococcus lactis NZ9000 expressing E6 oncoprotein. Microb Pathog (2019) 126:149–56. doi: 10.1016/j.micpath.2018.10.043 30391536

[122] Zhuang WR Wang Y Lei Y Zuo L Jiang A Wu G. Phytochemical engineered bacterial outer membrane vesicles for photodynamic effects promoted immunotherapy. Nano Lett (2022) 22(11):4491–500. doi: 10.1021/acs.nanolett.2c01280 35605283

[123] Liu Y Lu Y Ning B Su X Yang B Dong H. Intravenous delivery of living listeria monocytogenes elicits gasdmermin-dependent tumor pyroptosis and motivates anti-tumor immune response. ACS Nano (2022) 16(3):4102–15. doi: 10.1021/acsnano.1c09818 35262333

[124] Augustin LB Milbauer L Hastings SE Leonard AS Saltzman DA Schottel JL. Salmonella enterica typhimurium engineered for nontoxic systemic colonization of autochthonous tumors. J Drug Target (2021) 29(3):294–9. doi: 10.1080/1061186X.2020.1818759 32886538

[125] Kubiak AM Bailey TS Dubois LJ Theys J Lambin P. Efficient secretion of murine IL-2 from an attenuated strain of clostridium sporogenes, a novel delivery vehicle for cancer immunotherapy. Front Microbiol (2021) 12:669488. doi: 10.3389/fmicb.2021.669488 34168629 PMC8217651

[126] Chen Y Du M Yuan Z Chen Z Yan F. Spatiotemporal control of engineered bacteria to express interferon-gamma by focused ultrasound for tumor immunotherapy. Nat Commun (2022) 13(1):4468. doi: 10.1038/s41467-022-31932-x 35918309 PMC9345953

[127] Masuda H Nakamura T Noma Y Harashima H. Application of BCG-CWS as a systemic adjuvant by using nanoparticulation technology. Mol Pharm (2018) 15(12):5762–71. doi: 10.1021/acs.molpharmaceut.8b00919 30380885

[128] Chen Q Bai H Wu W Huang G Li Y Wu M. Bioengineering bacterial vesicle-coated polymeric nanomedicine for enhanced cancer immunotherapy and metastasis prevention. Nano Lett (2020) 20(1):11–21. doi: 10.1021/acs.nanolett.9b02182 31858807

[129] Johnson SA Ormsby MJ Wessel HM Hulme HE Bravo-Blas A McIntosh A. Monocytes mediate salmonella typhimurium-induced tumor growth inhibition in a mouse melanoma model. Eur J Immunol (2021) 51(12):3228–38. doi: 10.1002/eji.202048913 PMC9214623 34633664

[130] Gao J Wang S Dong X Wang Z. RGD-expressed bacterial membrane-derived nanovesicles enhance cancer therapy via multiple tumorous targeting. Theranostics (2021) 11(7):3301–16. doi: 10.7150/thno.51988 PMC7847689 33537088

[131] Geng Z Cao Z Liu R Liu K Liu J Tan W. Aptamer-assisted tumor localization of bacteria for enhanced biotherapy. Nat Commun (2021) 12(1):6584. doi: 10.1038/s41467-021-26956-8 34782610 PMC8593157

[132] Feng J Liu Y Pan X Jin F Wu L Chen J. Acid-directed electrostatic self-assembly generates charge-reversible bacteria for enhanced tumor targeting and low tissue trapping. ACS Appl Mater Interfaces (2022) 14(32):36411–24. doi: 10.1021/acsami.2c08684 35917371

[133] Guo F Das JK Kobayashi KS Qin QM Ficht TA Alaniz RC. Live attenuated bacterium limits cancer resistance to CAR-T therapy by remodeling the tumor microenvironment. J Immunother Cancer (2022) 10(1):e003760. doi: 10.1136/jitc-2021-003760 34987022 PMC8734016

[134] Leventhal DS Sokolovska A Li N Plescia C Kolodziej SA Gallant CW. Immunotherapy with engineered bacteria by targeting the STING pathway for anti-tumor immunity. Nat Commun (2020) 11(1):2739. doi: 10.1038/s41467-020-16602-0 32483165 PMC7264239

[135] Harimoto T Hahn J Chen YY Im J Zhang J Hou N. A programmable encapsulation system improves delivery of therapeutic bacteria in mice. Nat Biotechnol (2022) 40(8):1259–69. doi: 10.1038/s41587-022-01244-y PMC9371971 35301496

[136] Felgner S Kocijancic D Frahm M Heise U Rohde M Zimmermann K. Engineered salmonella enterica serovar typhimurium overcomes limitations of anti-bacterial immunity in bacteria-mediated tumor therapy. Oncoimmunology (2018) 7(2):e1382791. doi: 10.1080/2162402X.2017.1382791 29308303 PMC5749626

[137] Hu CW Chang YC Liu CH Yu YA Mou KY. Development of a TNF-alpha-mediated Trojan horse for bacteria-based cancer therapy. Mol Ther (2022) 30(7):2522–36. doi: 10.1016/j.ymthe.2022.04.008 PMC9263318 35440418

[138] Wang X Li X Onuma K Sogo Y Ohno T Ito A. Zn- and mg- containing tricalcium phosphates-based adjuvants for cancer immunotherapy. Sci Rep (2013) 3:2203. doi: 10.1038/srep02203 23857555 PMC3712317

[139] Zhu J Ke Y Liu Q Yang J Liu F Xu R. Engineered lactococcus lactis secreting Flt3L and OX40 ligand for in situ vaccination-based cancer immunotherapy. Nat Commun (2022) 13(1):7466. doi: 10.1038/s41467-022-35130-7 36463242 PMC9719518

[140] Lee HH Hong SH Rhee JH Lee SE. Optimal long peptide for flagellin-adjuvanted HPV E7 cancer vaccine to enhance tumor suppression in combination with anti-PD-1. Transl Cancer Res (2022) 11(6):1595–602. doi: 10.21037/tcr-21-2798 PMC9273650 35836530

[141] Mohabati Mobarez A Soleimani N Esmaeili SA Farhangi B. Nanoparticle-based immunotherapy of breast cancer using recombinant helicobacter pylori proteins. Eur J Pharm Biopharm (2020) 155:69–76. doi: 10.1016/j.ejpb.2020.08.013 32798667

[142] Singh M Quispe-Tintaya W Chandra D Jahangir A Venkataswamy MM Ng TW. Direct incorporation of the NKT-cell activator alpha-galactosylceramide into a recombinant listeria monocytogenes improves breast cancer vaccine efficacy. Br J Cancer (2014) 111(10):1945–54. doi: 10.1038/bjc.2014.486 PMC4229631 25314062

[143] Oladejo M Nguyen HM Silwal A Reese B Paulishak W Markiewski MM. Listeria-based immunotherapy directed against CD105 exerts anti-angiogenic and anti-tumor efficacy in renal cell carcinoma. Front Immunol (2022) 13:1038807. doi: 10.3389/fimmu.2022.1038807 36439126 PMC9692019

[144] Ni D Qing S Ding H Yue H Yu D Wang S. Biomimetically engineered demi-bacteria potentiate vaccination against cancer. Adv Sci (Weinh) (2017) 4(10):1700083. doi: 10.1002/advs.201700083 29051851 PMC5644226

[145] Han C Zhang X Pang G Zhang Y Pan H Li L. Hydrogel microcapsules containing engineered bacteria for sustained production and release of protein drugs. Biomaterials (2022) 287:121619. doi: 10.1016/j.biomaterials.2022.121619 35700622

[146] Ylosmaki E Fusciello M Martins B Feola S Hamdan F Chiaro J. Novel personalized cancer vaccine platform based on bacillus calmette-guerin. J Immunother Cancer (2021) 9(7):2707. doi: 10.1136/jitc-2021-002707 PMC8286790 34266884

[147] Cheng K Zhao R Li Y Qi Y Wang Y Zhang Y. Bioengineered bacteria-derived outer membrane vesicles as a versatile antigen display platform for tumor vaccination via plug-and-Display technology. Nat Commun (2021) 12(1):2041. doi: 10.1038/s41467-021-22308-8 33824314 PMC8024398

[148] Li Y Ma X Yue Y Zhang K Cheng K Feng Q. Rapid surface display of mRNA antigens by bacteria-derived outer membrane vesicles for a personalized tumor vaccine. Adv Mater (2022) 34(20):e2109984. doi: 10.1002/adma.202109984 35315546

[149] Jin C Liu Y Zhu J Xia T Zhang B Fei Y. Recombinant salmonella-based CEACAM6 and 4-1BBL vaccine enhances T-cell immunity and inhibits the development of colorectal cancer in rats: in vivo effects of vaccine containing 4-1BBL and CEACAM6. Oncol Rep (2015) 33(6):2837–44. doi: 10.3892/or.2015.3901 25872647

[150] Kim BJ Gong JR Kim GN Kim BR Lee SY Kook YH. Recombinant mycobacterium smegmatis with a pMyong2 vector expressing human immunodeficiency virus type I gag can induce enhanced virus-specific immune responses. Sci Rep (2017) 7:44776. doi: 10.1038/srep44776 28300196 PMC5353558

[151] Yoon W Park YC Kim J Chae YS Byeon JH Min SH. Application of genetically engineered salmonella typhimurium for interferon-gamma-induced therapy against melanoma. Eur J Cancer (2017) 70:48–61. doi: 10.1016/j.ejca.2016.10.010 27883926

[152] Aref NM Nasr M Osman R. Construction and immunogenicity analysis of nanoparticulated conjugate of heat-stable enterotoxin (STa) of enterotoxigenic escherichia coli. Int J Biol Macromol (2018) 106:730–8. doi: 10.1016/j.ijbiomac.2017.08.077 28823704

[153] Le Gouellec A Chauchet X Laurin D Aspord C Verove J Wang Y. A safe bacterial microsyringe for in vivo antigen delivery and immunotherapy. Mol Ther (2013) 21(5):1076–86. doi: 10.1038/mt.2013.41 PMC3666637 23531551

[154] Sebo P Osicka R Masin J. Adenylate cyclase toxin-hemolysin relevance for pertussis vaccines. Expert Rev Vaccines (2014) 13(10):1215–27. doi: 10.1586/14760584.2014.944900 25090574

[155] Budhu S Wolchok J Merghoub T. The importance of animal models in tumor immunity and immunotherapy. Curr Opin Genet Dev (2014) 24:46–51. doi: 10.1016/j.gde.2013.11.008 24657536 PMC4241684

[156] Staedtke V Roberts NJ Bai RY Zhou S. Clostridium novyi-NT in cancer therapy. Genes Dis (2016) 3(2):144–52. doi: 10.1016/j.gendis.2016.01.003 PMC6150096 30258882

[157] Dong W Zhang H Yin X Liu Y Chen D Liang X. Oral delivery of tumor microparticle vaccines activates NOD2 signaling pathway in ileac epithelium rendering potent antitumor T cell immunity. Oncoimmunology (2017) 6(3):e1282589. doi: 10.1080/2162402X.2017.1282589 28405506 PMC5384362

[158] Pan J Li X Shao B Xu F Huang X Guo X. Self-blockade of PD-L1 with bacteria-derived outer-membrane vesicle for enhanced cancer immunotherapy. Adv Mater (2022) 34(7):e2106307. doi: 10.1002/adma.202106307 34859919

[159] Sorieul C Papi F Carboni F Pecetta S Phogat S Adamo R. Recent advances and future perspectives on carbohydrate-based cancer vaccines and therapeutics. Pharmacol Ther (2022) 235:108158. doi: 10.1016/j.pharmthera.2022.108158 35183590

[160] Kim YH Lee JR Hahn MJ. Regulation of inflammatory gene expression in macrophages by epithelial-stromal interaction 1 (Epsti1). Biochem Biophys Res Commun (2018) 496(2):778–83. doi: 10.1016/j.bbrc.2017.12.014 29217193

[161] Noguera-Ortega E Guallar-Garrido S Julian E. Mycobacteria-based vaccines as immunotherapy for non-urological cancers. Cancers (Basel) (2020) 12(7):1802. doi: 10.3390/cancers12071802 32635668 PMC7408281

[162] Wood LM Paterson Y. Attenuated listeria monocytogenes: a powerful and versatile vector for the future of tumor immunotherapy. Front Cell Infect Microbiol (2014) 4:51. doi: 10.3389/fcimb.2014.00051 24860789 PMC4026700

[163] Marques-Neto LM Piwowarska Z Kanno AI Moraes L Trentini MM Rodriguez D. Thirty years of recombinant BCG: new trends for a centenary vaccine. Expert Rev Vaccines (2021) 20(8):1001–11. doi: 10.1080/14760584.2021.1951243 34224293

[164] Garris CS Wong JL Ravetch JV Knorr DA. Dendritic cell targeting with fc-enhanced CD40 antibody agonists induces durable antitumor immunity in humanized mouse models of bladder cancer. Sci Transl Med (2021) 13(594):eabd1346. doi: 10.1126/scitranslmed.abd1346 34011627 PMC8325152

[165] Toussaint B Chauchet X Wang Y Polack B Le Gouellec A. Live-attenuated bacteria as a cancer vaccine vector. Expert Rev Vaccines (2013) 12(10):1139–54. doi: 10.1586/14760584.2013.836914 24124876

[166] Chenal A Ladant D. Bioengineering of bordetella pertussis adenylate cyclase toxin for antigen-delivery and immunotherapy. Toxins (Basel) (2018) 10(7):302. doi: 10.3390/toxins10070302 30037010 PMC6070788

[167] Fan JY Huang Y Li Y Muluh TA Fu SZ Wu JB. Bacteria in cancer therapy: a new generation of weapons. Cancer Med (2022) 11(23):4457–68. doi: 10.1002/cam4.4799 PMC9741989 35522104

[168] Guo Y Song M Liu X Chen Y Xun Z Sun Y. Photodynamic therapy-improved oncolytic bacterial immunotherapy with FAP-encoding s. typhimurium. J Control Release (2022) 351:860–71. doi: 10.1016/j.jconrel.2022.09.050 36181917

[169] Shi L Liu X Li Y Li S Wu W Gao X. Living bacteria-based immuno-photodynamic therapy: metabolic labeling of clostridium butyricum for eradicating malignant melanoma. Adv Sci (Weinh) (2022) 9(14):e2105807. doi: 10.1002/advs.202105807 35277932 PMC9108598

[170] Zhang J Li Z Liu L Li L Zhang L Wang Y. Self-assembly catalase nanocomplex conveyed by bacterial vesicles for oxygenated photodynamic therapy and tumor immunotherapy. Int J Nanomed (2022) 17:1971–85. doi: 10.2147/IJN.S353330 PMC9076005 35530972

[171] Wu C Cui M Cai L Chen C Zhu X Wu Y. NIR-responsive photodynamic nanosystem combined with antitumor immune optogenetics bacteria for precise synergetic therapy. ACS Appl Mater Interfaces (2022) 14(11):13094–106. doi: 10.1021/acsami.2c01138 35262323

[172] Liu X Wu M Wang M Duan Y Phan C Qi G. Metabolically engineered bacteria as light-controlled living therapeutics for anti-angiogenesis tumor therapy. Mater Horiz (2021) 8(5):1454–60. doi: 10.1039/d0mh01582b 34846453

[173] Zheng P Fan M Liu H Zhang Y Dai X Li H. Self-propelled and near-Infrared-Phototaxic photosynthetic bacteria as photothermal agents for hypoxia-targeted cancer therapy. ACS Nano (2021) 15(1):1100–10. doi: 10.1021/acsnano.0c08068 33236885

[174] Xu W Ren D Yu Z Hou J Huang F Gan T. Bacteria-mediated tumor immunotherapy via photothermally-programmed PD1 expression. Nanoscale Adv (2022) 4(6):1577–86. doi: 10.1039/d1na00857a PMC9417531 36134371

[175] Yao Y Li J Li P Wang D Bao W Xiao Y. Bacterially synthesized tellurium nanorods for elimination of advanced malignant tumor by photothermal immunotherapy. Small (2022) 18(8):e2105716. doi: 10.1002/smll.202105716 34889048

[176] Sun R Liu M Lu J Chu B Yang Y Song B. Bacteria loaded with glucose polymer and photosensitive ICG silicon-nanoparticles for glioblastoma photothermal immunotherapy. Nat Commun (2022) 13(1):5127. doi: 10.1038/s41467-022-32837-5 36050316 PMC9433534

[177] Wu H Zhong D Zhang Z Wu Y Li Y Mao H. A bacteria-inspired morphology genetic biomedical material: self-propelled artificial microbots for metastatic triple negative breast cancer treatment. ACS Nano (2021) 15(3):4845–60. doi: 10.1021/acsnano.0c09594 33625212

[178] Yi X Zhou H Chao Y Xiong S Zhong J Chai Z. Bacteria-triggered tumor-specific thrombosis to enable potent photothermal immunotherapy of cancer. Sci Adv (2020) 6(33):eaba3546. doi: 10.1126/sciadv.aba3546 32851163 PMC7428325

[179] Uthaman S Pillarisetti S Hwang HS Mathew AP Huh KM Rhee JH. Tumor microenvironment-regulating immunosenescence-independent nanostimulant synergizing with near-infrared light irradiation for antitumor immunity. ACS Appl Mater Interfaces (2021) 13(4):4844–52. doi: 10.1021/acsami.0c20063 33486952

[180] Chen Q Huang G Wu W Wang J Hu J Mao J. A hybrid eukaryotic-prokaryotic nanoplatform with photothermal modality for enhanced antitumor vaccination. Adv Mater (2020) 32(16):e1908185. doi: 10.1002/adma.201908185 32108390

[181] Chen W He C Qiao N Guo Z Hu S Song Y. Dual drugs decorated bacteria irradiate deep hypoxic tumor and arouse strong immune responses. Biomaterials (2022) 286:121582. doi: 10.1016/j.biomaterials.2022.121582 35609407

[182] Zhuang Q Xu J Deng D Chao T Li J Zhang R. Bacteria-derived membrane vesicles to advance targeted photothermal tumor ablation. Biomaterials (2021) 268:120550. doi: 10.1016/j.biomaterials.2020.120550 33278684

[183] Safran H Leonard KL Perez K Vrees M Klipfel A Schechter S. Tolerability of ADXS11-001 lm-LLO listeria-based immunotherapy with mitomycin, fluorouracil, and radiation for anal cancer. Int J Radiat Oncol Biol Phys (2018) 100(5):1175–8. doi: 10.1016/j.ijrobp.2018.01.004 29722659

[184] Pei P Zhang Y Jiang Y Shen W Chen H Yang S. Pleiotropic immunomodulatory functions of radioactive inactivated bacterial vectors for enhanced cancer radio-immunotherapy. ACS Nano (2022) 16(7):11325–37. doi: 10.1021/acsnano.2c04982 35819107

[185] Wang W Xu H Ye Q Tao F Wheeldon I Yuan A. Systemic immune responses to irradiated tumours via the transport of antigens to the tumour periphery by injected flagellate bacteria. Nat BioMed Eng (2022) 6(1):44–53. doi: 10.1038/s41551-021-00834-6 35058589

[186] Levit R Savoy de Giori G de Moreno de LeBlanc A LeBlanc JG. Evaluation of vitamin-producing and immunomodulatory lactic acid bacteria as a potential co-adjuvant for cancer therapy in a mouse model. J Appl Microbiol (2021) 130(6):2063–74. doi: 10.1111/jam.14918 33128836

[187] Xu H Piao L Wu Y Liu X. IFN-gamma enhances the antitumor activity of attenuated salmonella-mediated cancer immunotherapy by increasing M1 macrophage and CD4 and CD8 T cell counts and decreasing neutrophil counts. Front Bioeng Biotechnol (2022) 10:996055. doi: 10.3389/fbioe.2022.996055 36246355 PMC9556780

[188] Wu Y Li Q Liu Y Li Y Chen Y Wu X. Targeting hypoxia for sensitization of tumors to apoptosis enhancement through supramolecular biohybrid bacteria. Int J Pharm (2021) 605:120817. doi: 10.1016/j.ijpharm.2021.120817 34166726

[189] Zhang W Liu J Li X Zheng Y Chen L Wang D. Precise chemodynamic therapy of cancer by trifunctional bacterium-based nanozymes. ACS Nano (2021) 15(12):19321–33. doi: 10.1021/acsnano.1c05605 34851608

[190] Bascuas T Moreno M Grille S Chabalgoity JA. Salmonella immunotherapy improves the outcome of CHOP chemotherapy in non-Hodgkin lymphoma-bearing mice. Front Immunol (2018) 9:7. doi: 10.3389/fimmu.2018.00007 29410666 PMC5787062

[191] Ektate K Munteanu MC Ashar H Malayer J Ranjan A. Chemo-immunotherapy of colon cancer with focused ultrasound and salmonella-laden temperature sensitive liposomes (thermobots). Sci Rep (2018) 8(1):13062. doi: 10.1038/s41598-018-30106-4 30166607 PMC6117346

[192] Moreo E Uranga S Pico A Gomez AB Nardelli-Haefliger D Del Fresno C. Novel intravesical bacterial immunotherapy induces rejection of BCG-unresponsive established bladder tumors. J Immunother Cancer (2022) 10(7):e004325. doi: 10.1136/jitc-2021-004325 35781395 PMC9252205

[193] Thomas SC Madaan T Kamble NS Siddiqui NA Pauletti GM Kotagiri N. Engineered bacteria enhance immunotherapy and targeted therapy through stromal remodeling of tumors. Adv Healthc Mater (2022) 11(2):e2101487. doi: 10.1002/adhm.202101487 34738725 PMC8770579

[194] Sivan A Corrales L Hubert N Williams JB Aquino-Michaels K Earley ZM. Commensal bifidobacterium promotes antitumor immunity and facilitates anti-PD-L1 efficacy. Science (2015) 350(6264):1084–9. doi: 10.1126/science.aac4255 PMC4873287 26541606

[195] Leon-Letelier RA Castro-Medina DI Badillo-Godinez O Tepale-Segura A Huanosta-Murillo E Aguilar-Flores C. Induction of progenitor exhausted tissue-resident memory CD8(+) T cells upon salmonella typhi porins adjuvant immunization correlates with melanoma control and anti-PD-1 immunotherapy cooperation. Front Immunol (2020) 11:583382. doi: 10.3389/fimmu.2020.583382 33240271 PMC7682137

[196] Jeong Y Kim GB Ji Y Kwak GJ Nam GH Hong Y. Dendritic cell activation by an e. coli-derived monophosphoryl lipid a enhances the efficacy of PD-1 blockade. Cancer Lett (2020) 472:19–28. doi: 10.1016/j.canlet.2019.12.012 31857157

[197] Ahmed SG Oliva G Shao M Wang X Mekalanos JJ Brenner GJ. Intratumoral injection of schwannoma with attenuated salmonella typhimurium induces antitumor immunity and controls tumor growth. Proc Natl Acad Sci USA (2022) 119(24):e2202719119. doi: 10.1073/pnas.2202719119 35675425 PMC9214496

[198] Meng F Li L Zhang Z Lin Z Zhang J Song X. Biosynthetic neoantigen displayed on bacteria derived vesicles elicit systemic antitumour immunity. J Extracell Vesicles (2022) 11(12):e12289. doi: 10.1002/jev2.12289 36468941 PMC9721206

[199] Zhao J Wang Y Wang J Lv M Zhou C Jia L. Lactobacillus kefiranofaciens ZW18 from kefir enhances the anti-tumor effect of anti-programmed cell death 1 (PD-1) immunotherapy by modulating the gut microbiota. Food Funct (2022) 13(19):10023–33. doi: 10.1039/d2fo01747d 36069328

[200] Wang L Cao Z Zhang M Lin S Liu J. Spatiotemporally controllable distribution of combination therapeutics in solid tumors by dually modified bacteria. Adv Mater (2022) 34(1):e2106669. doi: 10.1002/adma.202106669 34687102

[201] Abedi MH Yao MS Mittelstein DR Bar-Zion A Swift MB Lee-Gosselin A. Ultrasound-controllable engineered bacteria for cancer immunotherapy. Nat Commun (2022) 13(1):1585. doi: 10.1038/s41467-022-29065-2 35332124 PMC8948203

[202] Ghouse SM Vadrevu SK Manne S Reese B Patel J Patel B. Therapeutic targeting of vasculature in the premetastatic and metastatic niches reduces lung metastasis. J Immunol (2020) 204(4):990–1000. doi: 10.4049/jimmunol.1901208 31900334 PMC7012400

[203] Cronin M Le Boeuf F Murphy C Roy DG Falls T Bell JC. Bacterial-mediated knockdown of tumor resistance to an oncolytic virus enhances therapy. Mol Ther (2014) 22(6):1188–97. doi: 10.1038/mt.2014.23 PMC4048890 24569832

[204] Sun M Yang S Huang H Gao P Pan S Cheng Z. Boarding oncolytic viruses onto tumor-homing bacterium-vessels for augmented cancer immunotherapy. Nano Lett (2022) 22(12):5055–64. doi: 10.1021/acs.nanolett.2c00699 35583490

[205] Howard FHN Al-Janabi H Patel P Cox K Smith E Vadakekolathu J. Nanobugs as drugs: bacterial derived nanomagnets enhance tumor targeting and oncolytic activity of HSV-1 virus. Small (2022) 18(13):e2104763. doi: 10.1002/smll.202104763 35076148

[206] Aitken AS Roy DG Martin NT Sad S Bell JC Bourgeois-Daigneault MC. Brief communication; a heterologous oncolytic bacteria-virus prime-boost approach for anticancer vaccination in mice. J Immunother (2018) 41(3):125–9. doi: 10.1097/CJI.0000000000000208 PMC5895163 29293165

[207] Zhou S Gravekamp C Bermudes D Liu K. Tumour-targeting bacteria engineered to fight cancer. Nat Rev Cancer (2018) 18(12):727–43. doi: 10.1038/s41568-018-0070-z PMC6902869 30405213

[208] Griffin ME Hang HC. Improving immunotherapy response through the use of designer bacteria. Cancer Cell (2021) 39(12):1576–7. doi: 10.1016/j.ccell.2021.11.009 34906317

[209] Engineered bacteria increase l-arginine to improve immunotherapy response. Cancer Discov (2021) 11(12):2956. doi: 10.1158/2159-8290.CD-RW2021-146 34654704

[210] Darragh LB Oweida AJ Karam SD. Overcoming resistance to combination radiation-immunotherapy: a focus on contributing pathways within the tumor microenvironment. Front Immunol (2018) 9:3154. doi: 10.3389/fimmu.2018.03154 30766539 PMC6366147

[211] Shrimali R Ahmad S Berrong Z Okoev G Matevosyan A Razavi GSE. Agonist anti-GITR antibody significantly enhances the therapeutic efficacy of listeria monocytogenes-based immunotherapy. J Immunother Cancer (2017) 5(1):64. doi: 10.1186/s40425-017-0266-x 28807056 PMC5557467

[212] Pierce KM Miklavcic WR Cook KP Hennen MS Bayles KW Hollingsworth MA. The evolution and future of targeted cancer therapy: from nanoparticles, oncolytic viruses, and oncolytic bacteria to the treatment of solid tumors. Nanomater (Basel) (2021) 11(11):3018. doi: 10.3390/nano11113018 PMC8623458 34835785

[213] Li Y Zhang K Wu Y Yue Y Cheng K Feng Q. Antigen capture and immune modulation by bacterial outer membrane vesicles as in situ vaccine for cancer immunotherapy post-photothermal therapy. Small (2022) 18(14):e2107461. doi: 10.1002/smll.202107461 35152555

[214] Felgner S Frahm M Kocijancic D Rohde M Eckweiler D Bielecka A. aroA-deficient salmonella enterica serovar typhimurium is more than a metabolically attenuated mutant. mBio (2016) 7(5):e01220–16. doi: 10.1128/mBio.01220-16 PMC5013297 27601574

[215] Coutermarsh-Ott SL Broadway KM Scharf BE Allen IC. Effect of salmonella enterica serovar typhimurium VNP20009 and VNP20009 with restored chemotaxis on 4T1 mouse mammary carcinoma progression. Oncotarget (2017) 8(20):33601–13. doi: 10.18632/oncotarget.16830 PMC5464893 28431394

[216] Igarashi K Kawaguchi K Kiyuna T Miyake K Miyake M Singh AS. Tumor-targeting salmonella typhimurium A1-r is a highly effective general therapeutic for undifferentiated soft tissue sarcoma patient-derived orthotopic xenograft nude-mouse models. Biochem Biophys Res Commun (2018) 497(4):1055–61. doi: 10.1016/j.bbrc.2018.02.174 29481803

[217] Berger E Soldati R Huebener N Hohn O Stermann A Durmus T. Salmonella SL7207 application is the most effective DNA vaccine delivery method for successful tumor eradication in a murine model for neuroblastoma. Cancer Lett (2013) 331(2):167–73. doi: 10.1016/j.canlet.2012.12.026 23337288

[218] Qing S Lyu C Zhu L Pan C Wang S Li F. Biomineralized bacterial outer membrane vesicles potentiate safe and efficient tumor microenvironment reprogramming for anticancer therapy. Adv Mater (2020) 32(47):e2002085. doi: 10.1002/adma.202002085 33015871

[219] Augustin LB Milbauer L Hastings SE Leonard AS Saltzman DA Schottel JL. Virulence-attenuated salmonella engineered to secrete immunomodulators reduce tumour growth and increase survival in an autochthonous mouse model of breast cancer. J Drug Target (2021) 29(4):430–8. doi: 10.1080/1061186X.2020.1850739 33183080

[220] Massa PE Paniccia A Monegal A Marco A Rescigno M. Salmonella engineered to express CD20-targeting antibodies and a drug-converting enzyme can eradicate human lymphomas. Blood (2013) 122(5):705–14. doi: 10.1182/blood-2012-12-474098 23736700

[221] Chiang CY Chen YJ Wu CC Liu SJ Leng CH Chen HW. Efficient uptake of recombinant lipidated survivin by antigen-presenting cells initiates antigen cross-presentation and antitumor immunity. Front Immunol (2018) 9:822. doi: 10.3389/fimmu.2018.00822 29755461 PMC5932405

[222] Gao S Song J Chen F Wang Q Liu X Ren H. A novel immunotoxin - rCCK8PE38 targeting of CCK-r overexpressed colon cancers. J Drug Target (2015) 23(5):462–8. doi: 10.3109/1061186X.2015.1009073 25673265

[223] Xu M Zhou L Zhang Y Xie Z Zhang J Guo L. A fixed human umbilical vein endothelial cell vaccine with 2 tandem repeats of microbial HSP70 peptide epitope 407-426 as adjuvant for therapy of hepatoma in mice. J Immunother (2015) 38(7):276–84. doi: 10.1097/CJI.0000000000000091 26261891

[224] Sun M Ye H Shi Q Xie J Yu X Ling H. Both-In-One hybrid bacteria suppress the tumor metastasis and relapse via tandem-amplifying reactive oxygen species-immunity responses. Adv Healthc Mater (2021) 10(21):e2100950. doi: 10.1002/adhm.202100950 34541825

[225] Janku F Zhang HH Pezeshki A Goel S Murthy R Wang-Gillam A. Intratumoral injection of clostridium novyi-NT spores in patients with treatment-refractory advanced solid tumors. Clin Cancer Res (2021) 27(1):96–106. doi: 10.1158/1078-0432.CCR-20-2065 33046513

[226] Blanchard TG Czinn SJ. Identification of helicobacter pylori and the evolution of an efficacious childhood vaccine to protect against gastritis and peptic ulcer disease. Pediatr Res (2017) 81(1-2):170–6. doi: 10.1038/pr.2016.199 27701380

[227] Drake CG Pachynski RK Subudhi SK McNeel DG Antonarakis ES Bauer TM. Safety and preliminary immunogenicity of JNJ-64041809, a live-attenuated, double-deleted listeria monocytogenes-based immunotherapy, in metastatic castration-resistant prostate cancer. Prostate Cancer Prostatic Dis (2022) 25(2):219–28. doi: 10.1038/s41391-021-00402-8 PMC9184270 34257408

[228] Abei M Okumura T Fukuda K Hashimoto T Araki M Ishige K. A phase I study on combined therapy with proton-beam radiotherapy and in situ tumor vaccination for locally advanced recurrent hepatocellular carcinoma. Radiat Oncol (2013) 8:239. doi: 10.1186/1748-717X-8-239 24131485 PMC3854490

[229] Zhang C Zhang Z Wang L Han J Li F Shen C. Pseudomonas aeruginosa-mannose sensitive hemagglutinin injection treated cytokine-induced killer cells combined with chemotherapy in the treatment of malignancies. Int Immunopharmacol (2017) 51:57–65. doi: 10.1016/j.intimp.2017.08.003 28802902

[230] O’Ryan M Stoddard J Toneatto D Wassil J Dull PM. A multi-component meningococcal serogroup b vaccine (4CMenB): the clinical development program. Drugs (2014) 74(1):15–30. doi: 10.1007/s40265-013-0155-7 24338083 PMC3890039

[231] Hong J Dauros-Singorenko P Whitcombe A Payne L Blenkiron C Phillips A. Analysis of the escherichia coli extracellular vesicle proteome identifies markers of purity and culture conditions. J Extracell Vesicles (2019) 8(1):1632099. doi: 10.1080/20013078.2019.1632099 31275533 PMC6598517

[232] Zavan L Bitto NJ Johnston EL Greening DW Kaparakis-Liaskos M. Helicobacter pylori growth stage determines the size, protein composition, and preferential cargo packaging of outer membrane vesicles. Proteomics (2019) 19(1-2):e1800209. doi: 10.1002/pmic.201800209 30488570

[233] Yang S Zhao W Zhu M Hu H Wang W Zang Z. Tumor temporal proteome profiling reveals the immunological triple offensive induced by synthetic anti-cancer salmonella. Front Immunol (2021) 12:712936. doi: 10.3389/fimmu.2021.712936 34489962 PMC8417115

[234] van de Waterbeemd B Streefland M van der Ley P Zomer B van Dijken H Martens D. Improved OMV vaccine against neisseria meningitidis using genetically engineered strains and a detergent-free purification process. Vaccine (2010) 28(30):4810–6. doi: 10.1016/j.vaccine.2010.04.082 20483197

[235] Gerritzen MJH Maas RHW van den Ijssel J van Keulen L Martens DE Wijffels RH. High dissolved oxygen tension triggers outer membrane vesicle formation by neisseria meningitidis. Microb Cell Fact (2018) 17(1):157. doi: 10.1186/s12934-018-1007-7 30285743 PMC6171317

[236] van de Waterbeemd B Zomer G Kaaijk P Ruiterkamp N Wijffels RH van den Dobbelsteen GP. Improved production process for native outer membrane vesicle vaccine against neisseria meningitidis. PloS One (2013) 8(5):e65157. doi: 10.1371/journal.pone.0065157 23741478 PMC3669287

[237] Gerritzen MJH Stangowez L van de Waterbeemd B Martens DE Wijffels RH Stork M. Continuous production of neisseria meningitidis outer membrane vesicles. Appl Microbiol Biotechnol (2019) 103(23-24):9401–10. doi: 10.1007/s00253-019-10163-z PMC6867985 31676919

[238] Gao X Feng Q Wang J Zhao X. Bacterial outer membrane vesicle-based cancer nanovaccines. Cancer Biol Med (2022) 19(9):1290–300. doi: 10.20892/j.issn.2095-3941.2022.0452 PMC9500226 36172794

